# Cardiovascular and renal effectiveness of empagliflozin in routine care in East Asia: Results from the EMPRISE East Asia study

**DOI:** 10.1002/edm2.183

**Published:** 2020-09-16

**Authors:** Yutaka Seino, Dae Jung Kim, Daisuke Yabe, Elise Chia‐Hui Tan, Wook‐Jin Chung, Kyoung Hwa Ha, Masaomi Nangaku, Koichi Node, Riho Klement, Atsutaka Yasui, Wei‐Yu Lei, Sunwoo Lee, Moe H. Kyaw, Anouk Deruaz‐Luyet, Kimberly G. Brodovicz, Wayne H.‐H. Sheu, the EMPRISE East Asia study group

**Affiliations:** ^1^ Kansai Electric Power Medical Research Institute Kobe Japan; ^2^ Kansai Electric Power Hospital Osaka Japan; ^3^ Department of Endocrinology and Metabolism Ajou University School of Medicine Suwon Korea; ^4^ Department of Diabetes and Endocrinology Gifu University Graduate School of Medicine Gifu Japan; ^5^ Division of Metabolism and Molecular Medicine Kobe University Graduate School of Medicine Kobe Japan; ^6^ National Research Institute of Chinese Medicine Ministry of Health and Welfare Taipei Taiwan; ^7^ Institute of Hospital and Healthcare Administration National Yang‐Ming University Taipei Taiwan; ^8^ Department of Cardiovascular Medicine Gachon University Gil Medical Center Incheon Korea; ^9^ Division of Nephrology and Endocrinology The University of Tokyo Tokyo Japan; ^10^ Saga University Saga Japan; ^11^ EPID Research Tartu Estonia; ^12^ Nippon Boehringer Ingelheim Co., Ltd. Tokyo Japan; ^13^ Boehringer Ingelheim Taiwan Ltd. Taipei Taiwan; ^14^ Boehringer Ingelheim Korea Ltd Seoul Korea; ^15^ Boehringer Ingelheim Pharmaceuticals Inc Ridgefield CT USA; ^16^ Boehringer Ingelheim International GmbH Ingelheim Germany; ^17^ Division of Endocrinology and Metabolism Taichung Veterans General Hospital Taichung Taiwan

**Keywords:** database research, DPP‐IV inhibitor, SGLT2 inhibitor, heart failure

## Abstract

**Aim:**

To evaluate the effectiveness of empagliflozin in clinical practice in East Asia in the Empagliflozin Comparative Effectiveness and Safety (EMPRISE) East Asia study.

**Materials and methods:**

Data were obtained from the Medical Data Vision database (Japan), National Health Insurance Service database (South Korea) and National Health Insurance database (Taiwan). Patients aged ≥ 18 years with type 2 diabetes initiating empagliflozin or a dipeptidyl peptidase‐4 (DPP‐4) inhibitor were 1:1 propensity score (PS) matched into sequentially built cohorts of new users naïve to both drug classes. This design reduces confounding due to switching treatments, time lag and immortal time biases. Outcomes included hospitalization for heart failure (HHF), end‐stage renal disease (ESRD) and all‐cause mortality. Hazard ratios (HRs) and 95% CIs were estimated using Cox proportional models, controlling for > 130 baseline characteristics in each data source and pooled by random‐effects meta‐analysis.

**Results:**

Overall, 28 712 pairs of PS‐matched patients were identified with mean follow‐up of 5.7‐6.8 months. Compared with DPP‐4 inhibitors, the risk of HHF was reduced by 18% and all‐cause mortality was reduced by 36% with empagliflozin (HR 0.82; 95% CI 0.71‐0.94, and HR 0.64; 95% CI 0.50‐0.81, respectively). Reductions were consistent across countries, and in patients with and without baseline cardiovascular disease. ESRD was also significantly reduced with empagliflozin versus DPP‐4 inhibitors (HR 0.37; 95% CI 0.24‐0.58).

**Conclusions:**

Empagliflozin treatment was associated with reduced risk for HHF, all‐cause mortality and ESRD compared with DPP‐4 inhibitors in routine clinical practice in Japan, South Korea and Taiwan.

## Graphical abstract

The EMPRISE East Asia study used data from three healthcare databases in Japan, South Korea and Taiwan to evaluate the effects of empagliflozin on CV and renal outcomes in routine clinical practice. 28 712 pairs of propensity score‐matched patients were included. Empagliflozin treatment was associated with reduced risk for hospitalisation for heart failure, all‐cause mortality and end‐stage renal disease.

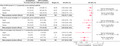

## 1 INTRODUCTION

According to the International Diabetes Federation (IDF), there are 163 million adults (aged 20‐79 years) with type 2 diabetes (T2D) in the Western Pacific Region, which is the highest number of any IDF region and represents 35% of all adults with diabetes worldwide. This number is expected to increase to 212 million by 2045.^1^

East Asian patients differ in pathophysiology and genetic susceptibility to T2D compared with Western patients, developing T2D with lower body mass index (BMI), higher visceral adiposity and greater pancreatic beta‐cell dysfunction.

Empagliflozin is a selective inhibitor of sodium‐glucose cotransporter‐2 (SGLT2)^2^ that has been approved for the treatment of T2D. In pooled analyses in Asian and East Asian patients, empagliflozin monotherapy or add‐on therapy improved glycaemic control, reduced body weight and blood pressure, and was well tolerated.^3^, ^4^ The EMPA‐REG OUTCOME trial showed that empagliflozin also provides heart and kidney benefits, in addition to the metabolic effects, in patients with T2D and established cardiovascular (CV) disease in addition to standard of care. Empagliflozin reduced the relative risk of CV death by 38%, all‐cause mortality by 32%, hospitalization for heart failure (HHF) by 35% and the incidence or worsening of nephropathy by 39% in patients with T2D and established CV disease.^5^, ^6^ In addition, CV, renal, and mortality outcomes were consistent among the overall trial population and patients from East Asian countries.^7^, ^8^ However, the effects of empagliflozin treatment have not been evaluated in routine clinical care in East Asia, in particular its use in a wider cohort of patients than included in the EMPA‐REG OUTCOME trial, such as patients with a broader spectrum of CV risk, including those without documented CV disease.

The EMPagliflozin CompaRative EffectIveness and SafEty (EMPRISE) study programme includes noninterventional studies of the effectiveness, safety, healthcare utilization and cost of care of empagliflozin in routine clinical practice in T2D patients across the CV risk continuum in East Asia, Europe and the US using comparable methodology.^9^ In the interim analysis of EMPRISE US (EUPAS20677, NCT03363464), empagliflozin was associated with a ~ 50% reduction in the risk of HHF compared with sitagliptin^10^ and lower risk of HHF^11^ and combined CV outcomes compared with dipeptidyl peptidase‐4 (DPP‐4) inhibitors, a class of glucose‐lowering agents that are used at a similar stage in the treatment pathway as empagliflozin and have neutral effects on CV outcomes.^12^

Here, we present the first analysis of data from EMPRISE East Asia (EUPAS27606, NCT03817463), evaluating the effectiveness of empagliflozin on CV and renal outcomes in routine clinical practice using data collected in Japan, South Korea and Taiwan.

## 2 MATERIALS AND METHODS

### 2.1 Study design

New users of empagliflozin or DPP‐4 inhibitors were identified from the Medical Data Vision (MDV) database in Japan (study period: December 2014 to April 2018), the National Health Insurance Service (NHIS) database in South Korea (May 2016 to December 2017), and the National Health Insurance claims database in Taiwan (May 2016 to December 2017). The MDV database covers more than 25 million patients from 374 acute hospitals while the NHIS and Taiwan databases are national databases. T2D diagnosis and clinical outcomes were identified using the 10th revision of the International Statistical Classification of Diseases and Related Health Problems (ICD‐10) in Japan and Korea and the both 9th (ICD‐9) and 10th revisions in Taiwan.

Eligible patients were aged 18 years or older at first prescription of empagliflozin or DPP‐4 inhibitor (see Table S1 for a list of included DPP‐4 inhibitors) and had a diagnosis of T2D prior to the index date (first prescription date), and no prescription of SGLT2 inhibitor or DPP‐4 inhibitor in the 12 months prior to the index date. Patients were not eligible for inclusion if they had a diagnosis of end‐stage renal disease (ESRD) in the 12 months prior to the index date, less than 12 months of data available prior to index data, or diagnosis of type 1 diabetes, secondary diabetes or gestational diabetes.

Cohorts of empagliflozin and DPP‐4 inhibitor initiators underwent 1:1 propensity score (PS) matching, adjusting for > 130‐149 covariates in each database. Covariates related to demographics, burden of comorbidities, diabetes‐related complications, diabetes medication, lifestyle factors, prior healthcare utilization and laboratory test results (Table S2). Postmatching covariate balance was assessed by absolute standardized differences (ASD), where ASD > 0.1 was considered to be a meaningful imbalance.^13^

Outcomes included HHF, all‐cause mortality (primary outcomes), and ESRD (secondary outcome). Definitions of outcomes are shown in Table S3.

In order to better capture HHF, two definitions were used: HHF‐specific and HHF‐broad. The intent of the HHF‐specific definition was to capture hospitalizations where the principal reason for the hospitalization was heart failure (HF). The intent of the HHF‐broad definition was to capture HF‐related hospitalizations where HF clinically contributed to hospitalization, although it may not have been the principal reason for hospitalization; therefore, the HHF‐broad definition includes HHF‐specific events. The HHF definitions in each country capture this intent while reflecting local coding practices.

In Japan, HHF‐specific was defined as an inpatient HF diagnosis that either required the most healthcare resources, triggered hospitalization or was coded as the main disease on the hospital claim.^14^ HHF‐broad was defined as any inpatient visit with an associated HF diagnosis code.

In Taiwan, HHF‐specific was defined as hospitalization when HF was the primary diagnosis at hospitalization while HHF‐broad was any hospitalization with a diagnosis of HF.

In South Korea, a single HHF definition was used (HF diagnosis code in any position of hospitalization), since the local coding practice is to record the underlying cause of HF (eg coronary heart disease) as the primary diagnosis.

Follow‐up for study outcomes started on the day after treatment initiation and continued in an ‘as‐treated’ approach until one of the following events occurred: study outcome, death, discontinuation of the initial drug, switch to another study drug (empagliflozin, any SGLT2 inhibitor, any DPP‐4 inhibitor), initiation of concomitant use of empagliflozin/SGLT2 inhibitor and a DPP‐4 inhibitor, or end of data availability.

### 2.2 Statistical analysis

Cox proportional hazards models were used to calculate HRs with 95% CIs separately for each country and pooled using random effects meta‐analysis models. Cox models were adjusted for any covariates that remained unbalanced (ASD > 0.1 after the PS matching. Heterogeneity and distribution were measured using the *I* ^2^ test and z test, respectively. Primary analyses were performed using an ‘as‐treated’ methodology. Sensitivity analyses of the main end‐points were performed using an intent‐to‐treat (ITT) methodology.

The HHF‐broad definition was used in the main analyses and the HHF‐specific definitions were used in sensitivity analyses. Sensitivity analyses for the HHF outcome included 1) HHF‐specific definition in Japan and Taiwan and the single definition for South Korea and 2) a ‘strict’ definition using the HHF‐specific definition in Japan and Taiwan and including only HHF diagnosis codes in the primary position in South Korea. Subgroup analyses were conducted for patients with and without CV disease and in patients receiving empagliflozin 10 mg/day. Within each subgroup, PS was re‐estimated and PS matching and analyses were performed again.

## 3 RESULTS

### 3.1 Patients

A total of 1 038 102 patients initiated either empagliflozin or a DPP‐4 inhibitor: 432 054 in Japan, 276 983 in South Korea and 329 065 in Taiwan.

**FIGURE 1:**
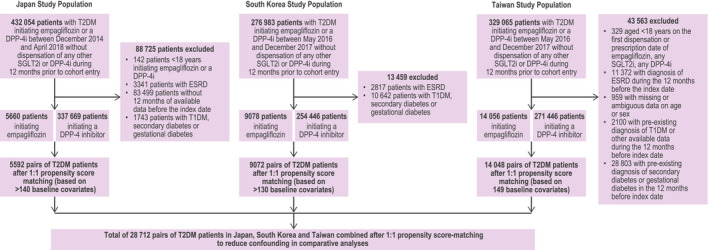
Flow chart of overall study population of empagliflozin versus DPP‐4 inhibitor population

A total of 28 712 pairs of PS‐matched patients were identified: 5592 pairs from Japan, 9072 from South Korea and 14 048 from Taiwan (Figure 1) including 98.8%, 99.9% and 99.9% of patients initiating empagliflozin and meeting inclusion criteria, respectively. Reasons for censoring for each outcome are provided as Table S4.

**FIGURE 2:**
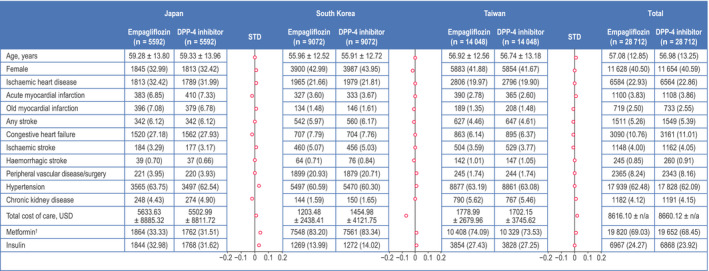
Baseline characteristics of patients initiating empagliflozin or DPP‐4 inhibitors after propensity score matching. ^†^Excluding fixed‐dose combinations with DPP‐4 and SGLT2 inhibitors. Data are n (%) of patients or mean ± SD. DPP‐4, dipeptidyl peptidase‐4; HbA_1c_, glycated haemoglobin; N/A, not available; STD, standardized difference, USD, US dollars

Mean and median (interquartile range) follow‐up, respectively, were 5.7 and 3.2 (0.9‐8.6) months in Japan, 6.8 and 5.7 (2.2‐10.1) months in South Korea, and 5.9 and 4.23 (1.87‐8.63) months in Taiwan. Within each country, baseline characteristics were similar after PS matching for patients treated with empagliflozin compared with those treated with DPP‐4 inhibitors (Figure 2, Table S2). Overall, mean age was approximately 57 years and the majority of patients (~60%) were male. With the exception of ischaemic heart disease and hypertension (~23% and ~ 62% of patients, respectively), the proportion of patients with CV comorbidities was low. The cohort from Japan was generally older with more comorbidities than the cohorts from South Korea and Taiwan; however, no heterogeneity was observed across the cohorts. At baseline, 68‐69% and ~ 24% of patients were receiving metformin and insulin, respectively. The most commonly used DPP‐4 inhibitors were sitagliptin, linagliptin and vildagliptin (Table S1).

### 3.2 Effect on patient outcomes

#### 3.2.1 Hospitalization for heart failure

**FIGURE 3:**
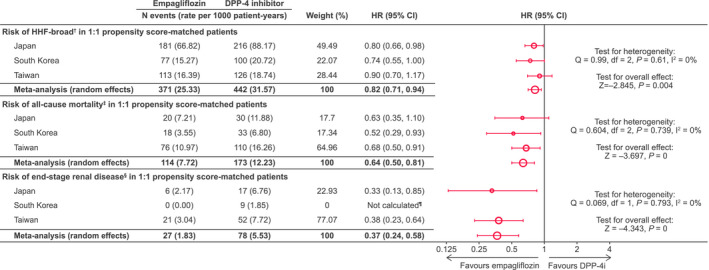
Risk of outcomes in 1:1 propensity score‐matched patients. ^†^ any hospitalization with a diagnosis of HF. ^‡^Death status was obtained via linking to the national death registries in Taiwan and South Korea, while death in Japan was captured through hospitalization discharge status. ^§^Estimated glomerular filtration rate < 15 mL/min/1.73 m^2^, at least 2 measurements separated by ≥ 30 days (≤12 months); ≥2 of the following diagnosis or procedure codes (either in/outpatient), separated by ≥ 30 days (stage 5 chronic kidney disease, end‐stage renal failure, haemodialysis, peritoneal dialysis); renal transplant. ^¶^HR not calculated due to 0 events in the empagliflozin group. CI, confidence interval; DPP‐4i, dipeptidyl peptidase‐4 inhibitor; HR, hazard ratio; HHF, hospitalization for heart failure

The risk of HHF was significantly reduced by 18% in empagliflozin initiators compared with DPP‐4 inhibitor initiators (HHF‐broad: HR 0.82; 95% CI 0.71‐0.94; (Figure 3). This risk reduction was consistent between countries (HHF‐broad: HR 0.80; 95% CI 0.66‐0.98 in Japan, 0.74; 95% CI 0.55‐1.00 in South Korea and 0.90; 95% CI 0.70‐1.17 in Taiwan; *I* ^2^ = 0%). In the sensitivity analysis using the HHF‐specific definition, empagliflozin was associated with a significant risk reduction of 21% compared with DPP‐4 inhibitors (overall HR 0.79; 95% CI 0.64‐0.97; *I* ^2^ = 0%; Figure S1).

#### 3.2.2 All‐cause mortality

All‐cause mortality was significantly reduced by 36% in patients receiving empagliflozin compared with DPP‐4 inhibitors (overall HR 0.64; 95% CI 0.50‐0.81) (Figure 3). This effect was comparable between countries (HR 0.63; 95% CI 0.35‐1.10 in Japan, 0.52; 95% CI 0.29‐0.93 in South Korea and 0.68; 95% CI 0.50‐0.91 in Taiwan; *I* ^2^ = 0%).

#### 3.2.3 End‐stage renal disease

A significant reduction in the risk of ESRD was observed with empagliflozin compared with the DPP‐4 inhibitor cohort (overall HR 0.37; 95% CI 0.24‐0.58), although this does not include South Korea as there were no events in the empagliflozin group in South Korea. Results were consistent between Japan and Taiwan (HR 0.33; 95% CI 0.13‐0.85 in Japan, HR 0.38; 95% CI 0.23‐0.64 in Taiwan; *I* ^2^ = 0%).

#### 3.2.4 Sensitivity analyses

Using the ITT methodology, risk reduction overall was 15% (HR 0.85; 95% CI 0.76‐0.95) for HHF‐broad, 28% (HR 0.72; 95% CI 0.51‐1.01) for all‐cause mortality and 60% (HR 0.40; 95% CI 0.28‐0.57) for ERSD (Figure S2).

#### 3.2.5 Subgroup analyses

**FIGURE 4:**
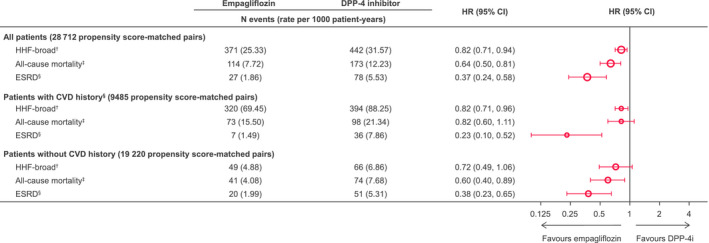
Risk of outcomes in subgroups of 1:1 propensity score‐matched patients. ^†^ any hospitalization with a diagnosis of HF. ^‡^Death status was obtained via linking to the national death registries in Taiwan and South Korea, while death in Japan was captured through hospitalization discharge status. ^§^Estimated glomerular filtration rate < 15 mL/min/1.73 m^2^, at least 2 measurements separated by ≥ 30 days (≤12 months); ≥2 of the following diagnosis or procedure codes (either in/outpatient), separated by ≥ 30 days (stage 5 chronic kidney disease, end‐stage renal failure, haemodialysis, peritoneal dialysis); renal transplant. ^¶^History of myocardial infarction, unstable angina, coronary atherosclerosis and other forms of chronic ischaemic heart disease, coronary procedure, heart failure, ischaemic or haemorrhagic stroke, transient ischaemic attack, peripheral arterial disease or surgery, lower extremity amputation. CI, confidence interval; CVD, cardiovascular disease; DPP‐4i, dipeptidyl peptidase‐4 inhibitor; ESRD, end‐stage renal disease; HR, hazard ratio; PS, propensity score

Results were consistent for all outcomes in those with and without CV disease at baseline (Figure 4, Figure S3). For example, the HR for HHF‐broad was 0.82 (95% CI 0.71‐0.96) in patients with CV disease and 0.72 (95% CI 0.49‐1.06) in patients without CV disease at baseline.

Findings in patients receiving 10 mg empagliflozin were consistent with the overall results (Table S5).

## 4 DISCUSSION

Type 2 diabetes is a risk factor for developing heart failure^15^ and is associated with worse outcomes for patients with heart failure, regardless of reduced and preserved ejection fraction.^16^ Although the incidence of heart failure was lower in East and Southeast Asian patients with T2D compared to white T2D patients,^17^, ^18^ the incidence is predicted to increase in Asia/East Asia.^19^ East Asian T2D patients have a higher risk of developing renal disease compared with other ethnicities ^17^, ^20^ and the incidence of ESRD in patients with T2D was estimated to be highest in East Asian and Pacific region compared with other regions.^21^

In patients from East Asia in the EMPRISE study, empagliflozin treatment in routine clinical practice was associated with significant reductions in the risk of HHF, all‐cause mortality and ESRD versus DPP‐4 inhibitors. These are the first data to describe the effectiveness of empagliflozin in routine clinical practice in Japan, South Korea and Taiwan.

The results of this study were consistent with those observed in EMPRISE US. In EMPRISE US, the risk of HHF‐broad was reduced by 49% with empagliflozin compared with sitagliptin (HR 0.51; 95% CI, 0.39‐0.68) and by 46% compared with DPP‐4 inhibitors (HR 0.56; 95% CI 0.43‐0.73).^10^ In the present study of patients from East Asia and in EMPRISE US, results were consistent in patients with or without baseline CV disease and across empagliflozin doses.

The results of this study also complement and expand upon the results of the EMPA‐REG OUTCOME trial to patients with T2D with or without CVD under routine clinical care.^6^ The reductions in HHF were comparable to those recorded in further analyses of HF outcomes in EMPA‐REG OUTCOME, where the HR versus placebo was 0.65 (95% CI 0.50‐0.85). While the exact mechanisms behind the benefits of empagliflozin on HF remain unclear, potential contributors include effects on natriuresis, reduction in preload, afterload and left ventricular wall stress, improved cardiac bioenergetics, improved kidney function and increased haematocrit levels.^22^, ^23^

The results of this study are also in line with those observed with SGLT2 inhibitors in other noninterventional studies, in which SGLT2 inhibitors were consistently associated with a reduced risk of HHF,^24^ E.^10^, ^25^ and all‐cause mortality compared with other glucose‐lowering drugs.^24^ This was reflective of the results from Western Europe and the USA seen in the original CVD‐REAL study.^26^ However, effects on all‐cause mortality in CVD‐REAL were lower than observed in this study and CV outcome trials of SGLT2 inhibitors such as the EMPA‐REG OUTCOME trial,^6^ CANVAS programme^27^ and DECLARE‐TIMI.^28^ The potential difference observed with EMPRISE East Asia could be due to the new user, active comparator design of EMPRISE, which minimizes biases such as immortal time bias by avoiding switching between SGLT2 inhibitors and DPP‐4 inhibitors, thereby avoiding an overestimate of the mortality rate in the comparator group.^9^

A significant reduction in the risk of ESRD was observed with empagliflozin compared to DPP‐4 inhibitors, although the number of events is low. This is particularly important given the high prevalence of ESRD in East Asia.^21^ This finding is supported by overall slower progression of kidney disease and lower rates of renal events in EMPA‐REG OUTCOME.^5^ A recent noninterventional study (CVD REAL 3), including some patients from Taiwan as well as data from databases in Israel, Italy, Japan and the UK also observed a consistent reduction in rate of ESRD in patients treated with SGLT2 inhibitors compared with other glucose‐lowering drugs.^29^ However, it is worth noting that, although baseline kidney‐related comorbidities were balanced between groups in this study, it cannot be ruled out that patients with poorer renal function may have received a DPP‐4 inhibitor instead of empagliflozin, thereby influencing the reduction in ESRD risk observed.

EMPRISE was designed to enhance balance across treatment groups and minimize chances of confounding and time‐related biases.^9^, ^30^, ^31^ This study has a number of additional strengths. Notably, it is reflective of routine clinical care in East Asia including active comparators that represent appropriate treatment alternatives to empagliflozin. The data included in this study were taken from three large, databases, which have been used in a number of real‐world evidence studies,^32^, ^33^S. E.,^34^Y. H.^35^, ^36^ In addition, the observed HHF rates in both EMPRISE US and this study were broadly similar to those observed in other studies using data from similar databases.^26^, ^37^ The propensity score methodology used in EMPRISE adjusted for > 130 covariates including baseline insulin and diabetes medication use, common comorbidities associated with diabetes, and health care utilization, all of which may be considered proxies for potential confounders not included in these databases such as diabetes severity and duration.^38^ Despite this, residual confounding for diabetes severity and duration as well as other potential confounders cannot be completely ruled out. As adherence to chronic therapy is known to be an issue in routine clinical care, primary analyses were conducted using an as‐treated approach, which improves comparability across the country analyses as this analytic approach does not depend upon patterns of nonadherence. In sensitivity analyses using an ITT methodology, risk reductions for all end‐points were comparable with those observed using the as‐treated methodology.

Potential limitations of this study include the low number of some events in some countries and subgroups. Residual confounding by some unmeasured characteristics is unlikely to be substantial but cannot be completely ruled out. While there is a limited possibility that patients may have previously received DPP‐4 inhibitors prior to the washout period, a one‐year washout period should suffice in clinical setting as glucose‐lowering agents are usually added on top of baseline therapy rather than switching. There are differences between healthcare and patient management between the countries in addition to differences between the datasets. Notably, the Japanese MDV database only included hospitalized patients. Baseline laboratory data including estimated glomerular filtration rate are not available except for a small subset of patients in the MDV database. In addition, the MDV database only captured in‐hospital death; despite this, all‐cause mortality was similar between Japan and Taiwan and South Korea where deaths were taken from national death databases. Length of follow‐up differed between countries due to when empagliflozin entered the market in each country. Differences in outcome definitions between the countries may have affected outcomes. Even though outcomes were defined using validated codes, there is a possibility that outcome misclassification may have affected these analyses. Follow‐up period was relatively short in this study (mean 5.7‐6.8 months), and there was the potential for some patients to have a very short follow‐up. However, risk reductions in HHF, mortality and composite renal outcome with empagliflozin were observed at very early and sustained throughout the EMPA‐REG outcome trial.^5^, ^6^, ^7^, ^8^ Therefore, the short follow‐up is not expected to affect the assessment. It is necessary to assess longer term effectiveness versus DPP‐4 inhibitors.

In conclusion, empagliflozin was associated with significant reductions in the risk of HHF, all‐cause mortality and ESRD compared with DPP‐4 inhibitors. Ongoing analyses of EMPRISE data from Asia, Europe and the US will include increasing numbers of patients and will provide further insights on the effectiveness, of empagliflozin in routine care in patients with T2D at the local, regional and global level.

## AUTHOR CONTRIBUTIONS

YS, DJK, DY, WJC, KHH, MN, KN, AY, WYL, SL, MHK, ADL, KGB and WHHS contributed to the concept and design of the analysis, interpretation of the data and critically revised the manuscript; KMH, ECHT and RK contributed to the concept and design of the analysis, analysis of data, interpretation of the data, provided statistical support and critically revised the manuscript; KGB is the guarantor of this work and, as such, had full access to all the data in the study and takes responsibility for the integrity of the data and the accuracy of the data analysis.

## Supporting information

Appendix S1

## Data Availability

The sponsor of the clinical trials (Boehringer Ingelheim) is committed to responsible sharing of clinical study reports, related clinical documents and patient‐level clinical study data. Researchers are invited to submit inquiries via the following website (https://trials.boehringer‐ingelheim.com/).
