#  

**DOI:** 10.1002/edm2.333

**Published:** 2023-03-20

In the article by Yutaka et al.,^1^ there were errors in the number of covariates for PS matching in several sections.

In the Materials and Methods section, ‘Hazard ratios (HRs) and 95% CIs were estimated using Cox proportional models, controlling for >130 baseline characteristics…’ should have been ‘Hazard ratios (HRs) and 95% CIs were estimated using Cox proportional models, controlling for ≥110 baseline characteristics…’.

In Figure 1, the baseline covariate numbers >140, >130 and 149 should have been 130, 110, and 166 for Japan, South Korea and Taiwan study population, respectively.

**FIGURE 1:**
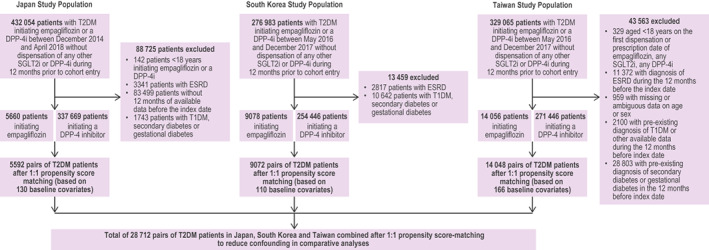
Flow chart of overall study population of empagliflozin versus DPP‐4 inhibitor population

In the study design sub‐section of Materials and Methods, ‘Cohorts of empagliflozin and DPP‐4 inhibitor initiators underwent 1:1 propensity score (PS) matching, adjusting for >130–149 covariates…’ should have been ‘Cohorts of empagliflozin and DPP‐4 inhibitor initiators underwent 1:1 propensity score (PS) matching, adjusting for 110–166 covariates…’.

In Figure 3, rate per 1000 patient‐years has been changed for Japan patients and, as the consequence, for meta‐analysis.

**FIGURE 3:**
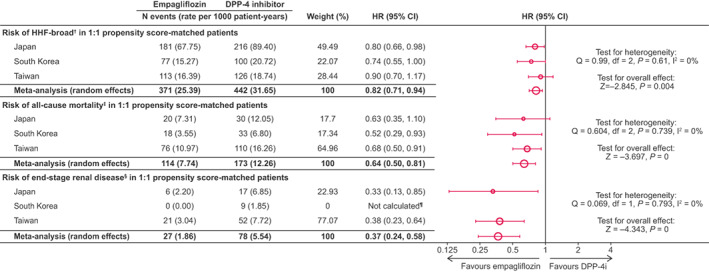
Risk of outcomes in 1:1 propensity score‐matched patients. † any hospitalization with a diagnosis of HF. ‡ Death status was obtained via linking to the national death registries in Taiwan and South Korea, while death in Japan was captured through hospitalizationdischarge status. § Estimated glomerular filtration rate < 15 mL/min/1.73 m2, at least 2 measurements separated by ≥ 30 days (≤12 months); ≥2 of the following diagnosis or procedure codes (either in/outpatient), separated by ≥ 30 days (stage 5 chronic kidney disease, endstage renal failure, haemodialysis, peritoneal dialysis); renal transplant. ¶HR not calculated due to 0 events in the empagliflozin group. CI, confidence interval; DPP‐4i, dipeptidyl peptidase‐4 inhibitor; HR, hazard ratio; HHF, hospitalization for heart failure

In the Discussion section, ‘The data included in this study were taken from three large, databases, which have been used in a number of real‐world evidence studies,^32,33^, S. E.,^34^ Y. H.^35,36^ ’ should have been ‘The data included in this study were taken from three large databases, which have been used in a number of real‐world evidence studies^32‐36’^.

In Figure 4, rate per 1000 patient‐years should have been changed for all patients, with CVD and without CVD history.

**FIGURE 4:**
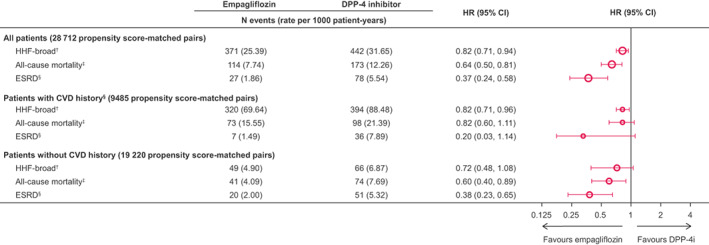
Risk of outcomes in subgroups of 1:1 propensity score‐matched patients. † any hospitalization with a diagnosis of HF. ‡ Death status was obtained via linking to the national death registries in Taiwan and South Korea, while death in Japan was captured through hospitalization discharge status. § Estimated glomerular filtration rate < 15 mL/min/1.73 m2, at least 2 measurements separated by ≥ 30 days (≤12 months); ≥2 of the following diagnosis or procedure codes (either in/outpatient), separated by ≥ 30 days (stage 5 chronic kidney disease, end‐stage renal failure, haemodialysis, peritoneal dialysis); renal transplant. ¶History of myocardial infarction, unstable angina, coronary atherosclerosis and other forms of chronic ischaemic heart disease, coronary procedure, heart failure, ischaemic or haemorrhagic stroke,transient ischaemic attack, peripheral arterial disease or surgery, lower extremity amputation. CI, confidence interval; CVD, cardiovascular disease; DPP‐4i, dipeptidyl peptidase‐4 inhibitor; ESRD, end‐stage renal disease; HR, hazard ratio; PS, propensity score

The following sentence under the same section, ‘The propensity score methodology used in EMPRISE adjusted for >130 covariates…’ should have been ‘The propensity score methodology used in EMPRISE adjusted for ≥110 covariates…’.

In Figure S1, S2 & S3, rate per 1000 patient‐years is changed for all patients, with CVD and without CVD history.

The authors apologize for these errors in the published article.

## Supporting information

supinfo
