# Supplementary material
Source: Endocrinol Diabetes Metab. 2023 Mar 20;6(3):e00333. doi: 10.1002/edm2.333 (PMC10164421; doi:10.1002/edm2.333)
Supplement: Supplementary file 1 — supinfo [file EDM2-6-e00333-s001.docx]

**Supplementary Appendix**

**EMPRISE East Asia Study Group**

Dr Ola Vedin (Boehringer Ingelheim AB, Stockholm, Sweden), Dr Lisette Koeneman (Eli Lilly and Company, Utrecht, Netherlands),Yusuke Taneda (Nippon Boehringer Ingelheim Co., Ltd. Tokyo, Japan), Jingbo Yi (Syneos Health Clinical K.K., Tokyo, Japan), Laura Saarelainen, Juha Mehtälä, Joni Oksanen (EPID Research, Espoo, Finland), Jinhee Lee (Department of Endocrinology and Metabolism, Ajou University School of Medicine, Suwon, Korea)

**FIGURE S1 Risk of HHF-specific in 1:1 propensity score-matched patients using a) HHF-specific sensitivity analysis^†^ and b) strict definition of HHF-specific**^‡^


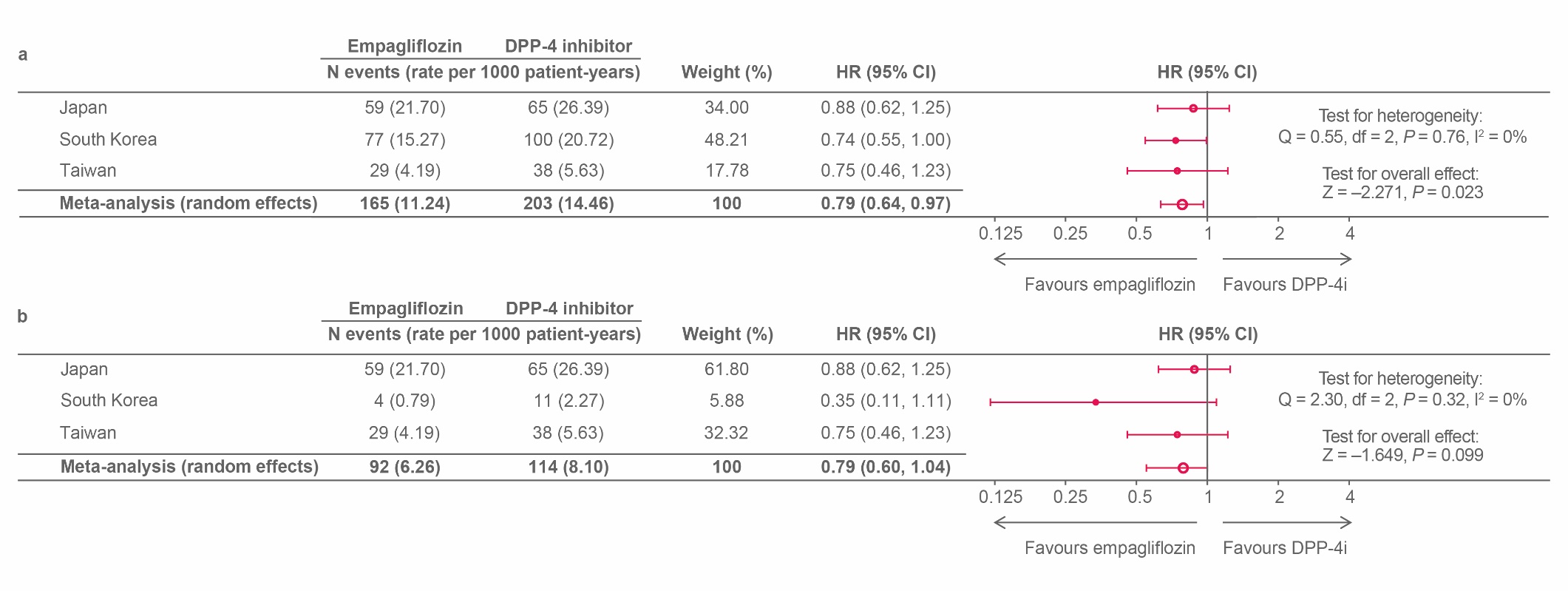


^†^HF as primary diagnosis code or main reason of admission for Japan and Taiwan, and hospitalization with any diagnosis code of HF in South Korea.

^‡^HF as primary diagnosis code or main reason of admission in all countries.

**FIGURE S2 Risk of outcomes in 1:1 propensity score-matched patients (ITT analysis)**


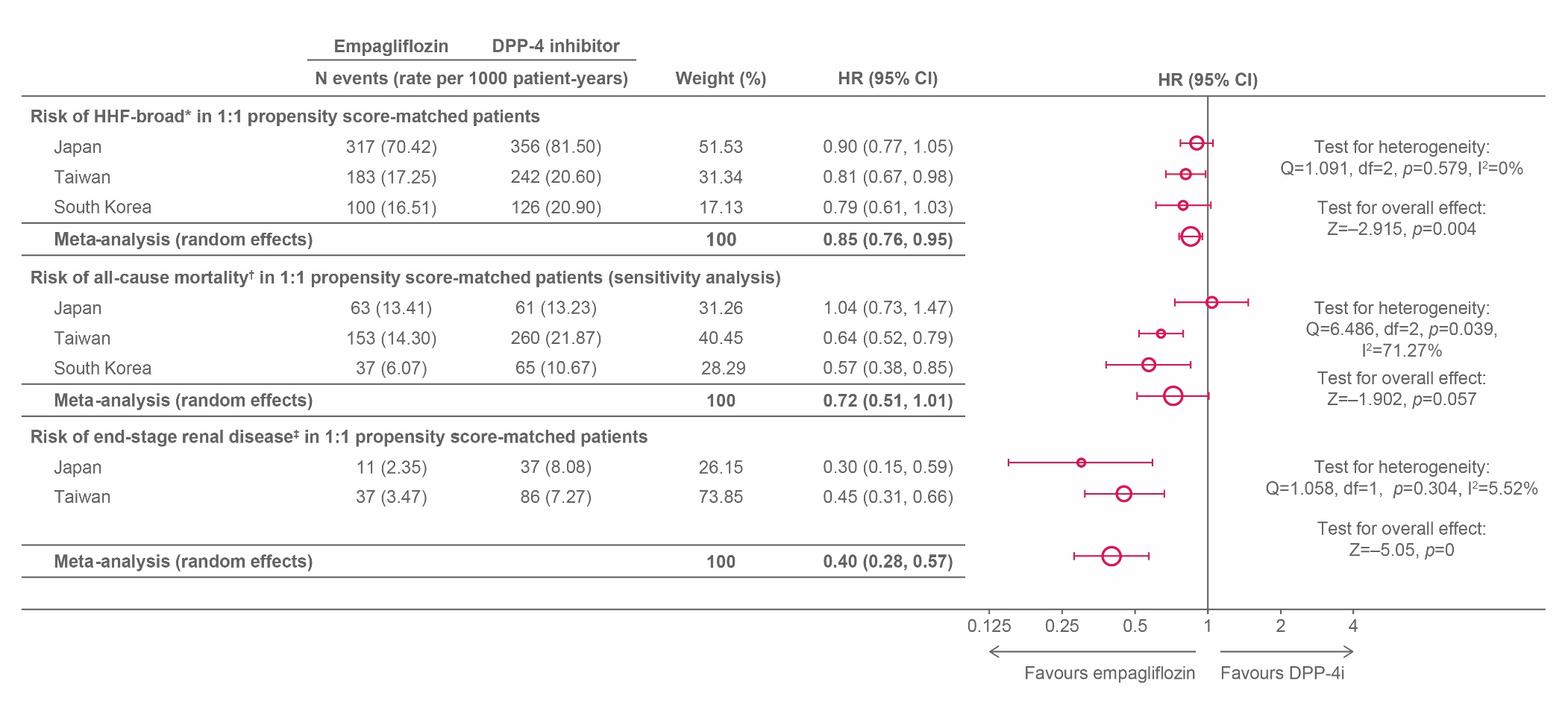


*Any heart failure diagnosis associated with healthcare encounters including hospitalisations and specialist outpatient and primary care encounters. ^†^Death status was obtained via linking to the national death registries in Taiwan and South Korea, while death in Japan was captured through hospitalisation discharge status. ^‡^Estimated glomerular filtration rate <15 ml/min/1.73 m2, at least 2 measurements separated by ≥30 days (≤12 months); ≥2 of the following diagnosis or procedure codes (either in/out-patient), separated by ≥30 days (stage 5 chronic kidney disease, end-stage renal failure, haemodialysis, peritoneal dialysis); renal transplant. CI, confidence interval; DPP-4i, dipeptidyl peptidase-4 inhibitor; HR, hazard ratio; HHF, hospitalisation for heart failure

**FIGURE S3 Risk of HHF-specific in subgroups of** **1:1 propensity score-matched patients using a) HHF-specific sensitivity analysis^†^ and b) strict definition of HHF-specific^‡^**


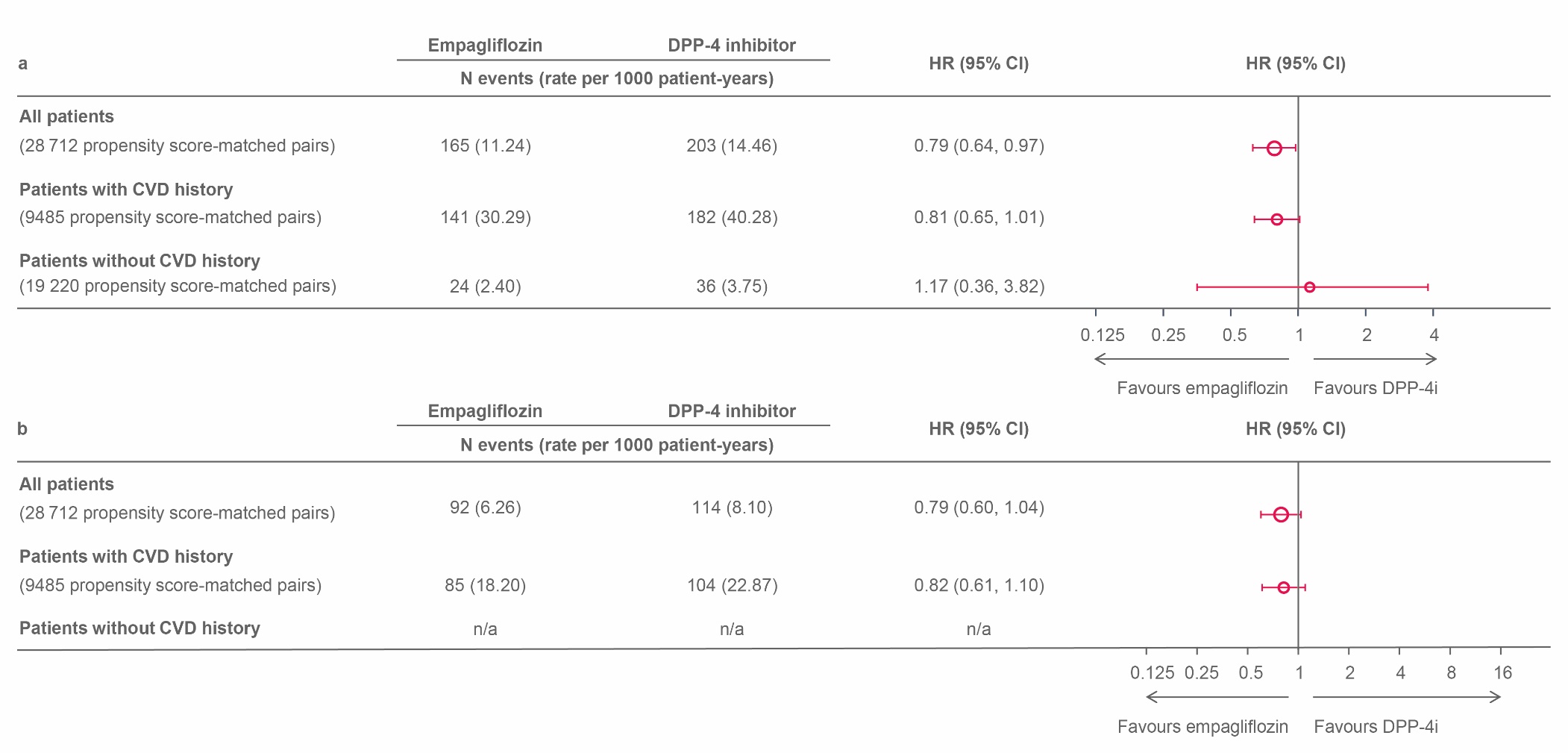


^†^HF as primary diagnosis code or main reason of admission for Japan and Taiwan, and hospitalization with any diagnosis code of HF in South Korea.
^‡^HF as primary diagnosis code or main reason of admission in all countries.

**TABLE S1 DPP-4 inhibitor use at baseline**

|  | **Japan** | **South Korea** | **Taiwan** |
| --- | --- | --- | --- |
| **DPP-4 inhibitor, n (%)** | **(N = 5592)** | **(N = 9072)** | **(N = 14048)** |
| Alogliptin | 213 (3.81) | 566 (6.24) | 79 (0.56) |
| Alogliptin + linagliptin + metformin | 1 (0.02) | 0 | 0 |
| Alogliptin + metformin | 138 (2.47) | 102 (1.12) | 136 (0.97) |
| Alogliptin + pioglitazone | 0 | 90 (0.99) | 0 |
| Alogliptin + sitagliptin | 1 (0.02) | 0 | 0 |
| Alogliptin + teneligliptin | 1 (0.02) | 0 | 0 |
| Anagliptin | 44 (0.79) | 233 (2.57) | 0 |
| Anagliptin + metformin | 39 (0.70)0 | 140 (1.54) | 0 |
| Evogliptin | 0 | 279 (3.08) | 0 |
| Evogliptin + Metformin | 0 | 109 (1.20) | 0 |
| Gemigliptin | 0 | 1025 (11.30) | 0 |
| Gemigliptin + Metformin | 0 | 785 (8.65) | 0 |
| Gemigliptin + Rosuvastatin | 0 | 1 (0.01) | 0 |
| Linagliptin | 834 (14.91) | 1305 (14.38) | 1589 (11.31) |
| Linagliptin + Metformin | 227 (4.06) | 600 (6.61) | 2651 (18.87) |
| Linagliptin + Vildagliptin | 1 (0.02) | 0 | 0 |
| Linagliptin + Vildagliptin + Metformin | 1 (0.02) | 0 | 0 |
| Omarigriptin | 51 (0.91) | 0 | 0 |
| Omarigriptin + Metformin | 33 (0.59) | 0 | 0 |
| Saxagliptin | 103 (1.84) | 223 (2.46) | 366 (2.61) |
| Saxagliptin + Metformin | 57 (1.02) | 221 (2.44) | 1051 (7.48) |
| Sitagliptin | 1281 (22.91) | 1032 (11.38) | 988 (7.03) |
| Sitagliptin + Metformin | 696 (12.45) | 901 (9.93) | 3730 (26.55) |
| Teneligliptin | 513 (9.17) | 653 (7.20) | 0 |
| Teneligliptin + Metformin | 202 (3.61) | 290 (3.20) | 0 |
| Trelagliptin | 64 (1.14) | 0 | 0 |
| Trelagliptin + Metformin | 24 (0.43) | 0 | 0 |
| Vildagliptin | 450 (8.05) | 237 (2.61) | 795 (5.66) |
| Vildagliptin + Metformin | 618 (11.05) | 280 (3.09) | 2663 (18.96) |

**Supplementary Table 2 Post-propensity matching baseline values of covariates**

|  | **Japan** | | | **South Korea** | | | **Taiwan** | | | **Total** | |
| --- | --- | --- | --- | --- | --- | --- | --- | --- | --- | --- | --- |
| **Covariate** | **EMPA** | **DPP-4 inhibitor** | **ASD** | **EMPA** | **DPP-4 inhibitor** | **ASD** | **EMPA** | **DPP-4 inhibitor** | **ASD** | **EMPA** | **DPP-4 inhibitor** |
| Number of patients | 5592 | 5592 |  | 9072 | 9072 |  | 14 048 | 14 048 |  | 28 712 | 28 712 |
| **Baseline characteristics** | | | | | | | | | | | |
| Age at index, mean (SD) | 59.28 (13.80) | 59.33 (13.96) | 0.00 | 55.96 (12.52) | 55.91 (12.72) | 0.00 | 56.92 (12.56) | 56.74 (13.18) | 0.01 | 57.08 (12.85) | 56.98 (13.25) |
| Age subcategory*,* n (%) | | | | | | | | | | | |
| 18–54 | 2047 (36.61) | 2049 (36.64) | n/a | 3941 (43.44) | 3944 (43.47) | n/a | 5882 (41.87) | 6076 (43.25) | n/a | 11 870 (41.34) | 12 069 (42.03) |
| 55–64 | 1336 (23.89) | 1278 (22.85) | n/a | 2915 (32.13) | 2898 (31.94) | n/a | 4454 (31.71) | 4228 (30.10) | n/a | 8705 (30.32) | 8404 (29.27) |
| 65–74 | 1458 (26.07) | 1501 (26.84) | n/a | 1570 (17.31) | 1585 (17.47) | n/a | 2708 (19.28) | 2593 (18.46) | n/a | 5736 (19.98) | 5679 (19.78) |
| ≥75 | 751  (13.43) | 764  (13.66) | n/a | 646 (7.12) | 645 (7.11) | n/a | 1004 (7.15) | 1151  (8.19) | n/a | 2401 (8.36) | 2560 (8.92) |
| Sex*,* n (%) | | | | | | | | | | | |
| Female | 1845 (32.99) | 1813 (32.42) | 0.01 | 3900 (42.99) | 3987 (43.95) | 0.02 | 5883 (41.88) | 5854 (41.67) | 0.00 | 11 628 (40.50) | 11 654 (40.59) |
| Socioeconomic status*,* n (%)^†^ | | | | | | | | | | | |
| Low | n/a | n/a | n/a | 1982 (21.85) | 1990 (21.94) | 0.00 | n/a | n/a | n/a | n/a | n/a |
| Intermediate | n/a | n/a | n/a | 3096 (34.13) | 3049 (33.61) | n/a | n/a | n/a | n/a | n/a | n/a |
| High | n/a | n/a | n/a | 3497 (38.55) | 3529 (38.90) | n/a | n/a | n/a | n/a | n/a | n/a |
| Year of index date*,* n (%) | | | | | | | | | | | |
| 2015 | 50 (0.89) | 50 (0.89) | 0.00 | 0 (0.00) | 0 (0.00) | 0.01 | 0 (0.00) | 0 (0.00) | 0.01 | 50 (0.17) | 50 (0.17) |
| 2016 | 1344 (24.03) | 1342 (24.00) | n/a | 2241 (24.70) | 2218 (24.45) | 0.01 | 4826 (34.35) | 4786 (34.07) | n/a | 8411 (29.29) | 8346 (29.07) |
| 2017 | 3049 (54.52) | 3052 (54.58) | n/a | 6831 (75.30) | 6854 (75.55) | n/a | 9222 (65.65) | 9262 (65.93) | n/a | 19 102 (66.53) | 19 168 (66.76) |
| 2018 | 1149 (20.55) | 1148 (20.53) | n/a | 0 (0.00) | 0 (0.00) | n/a | 0 (0.00) | 0 (0.00) | n/a | 1149 (3.92) | 1148 (3.91) |
| **Lifestyle variables** | | | | | | | | | | | |
| Obesity*,* n (%)^‡^ |  |  |  |  |  |  |  |  |  |  |  |
| Yes | 245 (4.38) | 236 (4.22) | 0.01 | n/a | n/a | n/a | 484 (3.45) | 486 (3.46) | 0.00 | n/a | n/a |
| No | 5347 (95.62) | 5356 (95.78) | n/a | n/a | n/a | n/a | 13 564 (96.55) | 13 562 (96.54) | n/a | n/a | n/a |
| Overweight*,* n (%)^‡^ |  |  |  |  |  |  |  |  |  |  |  |
| Yes | n/a | n/a | n/a | n/a | n/a | n/a | 5 (0.04) | 5 (0.04) | 0.00 | n/a | n/a |
| No | n/a | n/a | n/a | n/a | n/a | n/a | 14 043 (99.96) | 14 043 (99.96) | n/a | n/a | n/a |
| Smoker*,* n (%) |  |  |  |  |  |  |  |  |  |  |  |
| Yes | 24 (0.43) | 21 (0.38) | 0.01 | n/a | n/a | n/a | 282 (2.01) | 276 (1.96) | 0.00 | n/a | n/a |
| No | 5568 (99.57) | 5571 (99.62) |  | n/a | n/a | n/a | 13 766 (97.99) | 13 772 (98.04) | n/a | n/a | n/a |
| Alcohol abuse or dependence*,* n (%) |  |  |  |  |  |  |  |  |  |  |  |
| Yes | 7 (0.13) | 9 (0.16) | 0.01 | n/a | n/a | n/a | 133 (0.95) | 127 (0.90) | 0.01 | n/a | n/a |
| No | 5585 (99.87) | 5583 (99.84) | n/a | n/a | n/a | n/a | 13 915 (99.05) | 13 921 (99.10) | n/a | n/a | n/a |
| Drug abuse or dependence*,* n (%) |  |  |  |  |  |  |  |  |  |  |  |
| Yes | 0 (0.00) | 0 (0.00) | n/a | n/a | n/a | n/a | 10 (0.07) | 13 (0.09) | 0.01 | n/a | n/a |
| No | 5592 (100.00) | 5592 (100.00) | n/a | n/a | n/a | n/a | 14 038 (99.93) | 14 035 (99.91) | n/a | n/a | n/a |
| **Diabetes complications** | | | | | | | | | | | |
| Diabetic retinopathy*,* n (%) | 819 (14.65) | 738 (13.20) | 0.04 | 1791 (19.74) | 1760 (19.40) | 0.01 | 1364 (9.71) | 1347 (9.59) | 0.00 | 3974 (13.84) | 3845 (13.39) |
| Diabetes with other ophthalmic manifestations*,* n (%) | 121 (2.16) | 115 (2.06) | 0.01 | 2254 (24.85) | 2286 (25.20) | 0.01 | 174 (1.24) | 155 (1.10) | 0.01 | 2549 (8.88) | 2556 (8.90) |
| Retinal detachment, vitreous haemorrhage, vitrectomy*,* n (%) | 64 (1.14) | 66 (1.18) | 0.00 | 189 (2.08) | 191 (2.11) | 0.00 | 107 (0.76) | 109 (0.78) | 0.00 | 360 (1.25) | 366 (1.28) |
| Retinal laser coagulation therapy*,* n (%) | 59 (1.06) | 55 (0.98) | 0.01 | 130 (1.43) | 132 (1.46) | 0.00 | 176 (1.25) | 162 (1.15) | 0.01 | 365 (1.27) | 349 (1.22) |
| Diabetic neuropathy*,* n (%) | 175 (3.13) | 196 (3.51) | 0.02 | 1522 (16.78) | 1497 (16.50) | 0.01 | 942 (6.71) | 946 (6.73) | 0.00 | 2639 (9.19) | 2639 (9.19) |
| Diabetic nephropathy, n (%) | 432 (7.73) | 423 (7.56) | 0.01 | 1294 (14.26) | 1310 (14.44) | 0.01 | 2754 (19.60) | 2721 (19.37) | 0.01 | 4480 (15.60) | 4454 (15.51) |
| Hypoglycaemia*,* n (%) | 38 (0.68) | 41 (0.73) | 0.01 | 99 (1.09) | 109 (1.20) | 0.01 | 70 (0.5) | 66 (0.47) | 0.00 | 207 (0.72) | 216 (0.75) |
| Hyperglycaemia*,* n (%) | 47 (0.84) | 52 (0.93) | 0.01 | n/a | n/a | n/a | 104 (0.74) | 104 (0.74) | 0.00 | n/a | n/a |
| Disorders of fluid electrolyte and acid-base balance*,* n (%) | 479 (8.57) | 463 (8.28) | 0.01 | 649 (7.15) | 648 (7.14) | 0.00 | 314 (2.24) | 321 (2.29) | 0.00 | 1442 (5.02) | 1432 (4.99) |
| Diabetic ketoacidosis*,* n (%) | 0 (0.00) | 0 (0.00) |  | 35 (0.39) | 40 (0.44) | 0.01 | 30 (0.21) | 19 (0.14) | 0.02 | n/a | n/a |
| Hyperosmolar hyperglycaemic nonketotic syndrome*,* n (%) | 3 (0.05) | 4 (0.07) | 0.01 | 63 (0.69) | 59 (0.65) | 0.01 | 89 (0.63) | 94 (0.67) | 0.00 | 155 (0.54) | 157 (0.55) |
| Diabetes with peripheral circulatory disorders*,* n (%) | 43 (0.77) | 41 (0.73) | 0.00 | 48 (0.53) | 43 (0.47) | 0.01 | 176 (1.25) | 173 (1.23) | 0.00 | 267 (0.93) | 257 (0.90) |
| Diabetic foot*,* n (%) | 41 (0.73) | 34 (0.61) | 0.02 | n/a | n/a | n/a | 26 (0.19) | 28 (0.20) | 0.00 | n/a | n/a |
| Gangrene*,* n (%) | 16 (0.29) | 13 (0.23) | 0.01 | n/a | n/a | n/a | 20 (0.14) | 18 (0.13) | 0.00 | n/a | n/a |
| Lower extremity amputation*,* n (%) | 5 (0.09) | 4 (0.07) | 0.01 | 7 (0.08) | 7 (0.08) | 0.00 | 3 (0.02) | 0 (0.00) | 0.02 | 15 (0.05) | 46 (0.04) |
| Osteomyelitis*,* n (%) | 11 (0.20) | 10 (0.18) | 0.00 | n/a | n/a | n/a | 33 (0.23) | 35 (0.25) | 0.00 | n/a | n/a |
| Skin infections*,* n (%) | 233 (4.17) | 226 (4.04) | 0.01 | n/a | n/a | n/a | 1922 (13.68) | 1878 (13.37) | 0.01 | n/a | n/a |
| Erectile dysfunction*,* n (%) | 5 (0.09) | 3 (0.05) | 0.01 | n/a | n/a | n/a | 97 (0.69) | 103 (0.73) | 0.00 | n/a | n/a |
| Diabetes with unspecified complication*,* n (%) | 3 (0.05) | 2 (0.04) | 0.01 | n/a | n/a | n/a | 1831 (13.03) | 1808 (12.87) | 0.00 | n/a | n/a |
| Diabetes mellitus without mention of complications*,* n (%) | 1443 (25.80) | 1473 (26.34) | 0.01 | n/a | n/a | n/a | 10 586 (75.36) | 10 601 (75.46) | 0.00 | n/a | n/a |
| **Other comorbidities** | | | | | | | | | | | |
| Hypertension*,* n (%) | 3565 (63.75) | 3497 (62.54) | 0.03 | 5497 (60.59) | 5470 (60.30) | 0.01 | 8877 (63.19) | 8861 (63.08) | 0.00 | 17 939 (62.48) | 17 828 (62.09) |
| Hyperlipidaemia*,* n (%) | 3347 (59.85) | 3292 (58.87) | 0.02 | 7893 (87.00) | 7900 (87.08) | 0.00 | 9515 (67.73) | 9608 (68.39) | 0.01 | 20 755 (72.29) | 20 800 (72.44) |
| Ischaemic heart disease*,* n (%) | 1813 (32.42) | 1789 (31.99) | 0.01 | 1965 (21.66) | 1979 (21.81) | 0.00 | 2806 (19.97) | 2796 (19.90) | 0.00 | 6584 (22.93) | 6564 (22.86) |
| Acute myocardial infarction*,* n (%) | 383 (6.85) | 410 (7.33) | 0.02 | 327 (3.60) | 333 (3.67) | 0.00 | 390 (2.78) | 365 (2.60) | 0.01 | 1100 (3.83) | 1108 (3.86) |
| Acute coronary syndrome/unstable angina*,* n (%) | 268 (4.79) | 262 (4.69) | 0.01 | 404 (4.45) | 425 (4.68) | 0.01 | 833 (5.93) | 811 (5.77) | 0.01 | 1505 (5.24) | 1498 (5.22) |
| Old myocardial infarction*,* n (%) | 396 (7.08) | 379 (6.78) | 0.01 | 134 (1.48) | 146 (1.61) | 0.01 | 189 (1.35) | 208 (1.48) | 0.01 | 719 (2.50) | 733 (2.55) |
| Stable angina*,* n (%) | 1222 (21.85) | 1175 (21.01) | 0.02 | 423 (4.66) | 461 (5.08) | 0.02 | 748 (5.32) | 726 (5.17) | 0.01 | 2393 (8.33) | 2362 (8.23) |
| Coronary atherosclerosis and other forms of chronic ischaemic heart disease*,* n (%) | 275 (4.92) | 290 (5.19) | 0.01 | 726 (8.00) | 731 (8.06) | 0.00 | 2174 (15.48) | 2203 (15.68) | 0.01 | 3175 (11.06) | 3224 (11.23) |
| Other atherosclerosis*,* n (%) | 426 (7.62) | 394 (7.05) | 0.02 | 652 (7.19) | 626 (6.90) | 0.01 | 1288 (9.17) | 1310 (9.33) | 0.01 | 2366 (8.24) | 2330 (8.12) |
| Previous cardiac procedure (CABG or PTCA or Stent)*,* n (%) | 409 (7.31) | 443 (7.92) | 0.02 | 206 (2.27) | 205 (2.26) | 0.00 | 459 (3.27) | 484 (3.45) | 0.01 | 1074 (3.74) | 1132 (3.94) |
| History of CABG or PTCA*,* n (%) | 17 (0.30) | 14 (0.25) | 0.01 | 356 (3.92) | 351 (3.87) | 0.00 | 24 (0.17) | 27 (0.19) | 0.00 | 397 (1.38) | 392 (1.37) |
| Any stroke*,* n (%) | 342 (6.12) | 342 (6.12) | 0.00 | 542 (5.97) | 560 (6.17) | 0.01 | 627 (4.46) | 647 (4.61) | 0.01 | 1511 (5.26) | 1549 (5.39) |
| Ischaemic stroke (w and w/o mention of cerebral infarction)*,* n (%) | 184 (3.29) | 177 (3.17) | 0.01 | 460 (5.07) | 456 (5.03) | 0.00 | 504 (3.59) | 529 (3.77) | 0.01 | 1148 (4.00) | 1162 (4.05) |
| Haemorrhagic stroke*,* n (%) | 39 (0.70) | 37 (0.66) | 0.00 | 64 (0.71) | 76 (0.84) | 0.02 | 142 (1.01) | 147 (1.05) | 0.00 | 245 (0.85) | 260 (0.91) |
| TIA*,* n (%) | 18 (0.32) | 16 (0.29) | 0.01 | 190 (2.09) | 198 (2.18) | 0.01 | 209 (1.49) | 220 (1.57) | 0.01 | 417 (1.45) | 434 (1.51) |
| Other cerebrovascular disease*,* n (%) | 81 (1.45) | 71 (1.27) | 0.02 | 545 (6.01) | 535 (5.90) | 0.00 | 391 (2.78) | 379 (2.70) | 0.00 | 1,017 (3.54) | 985 (3.43) |
| Late effects of cerebrovascular disease*,* n (%) | 209 (3.74) | 185 (3.31) | 0.02 | 223 (2.46) | 229 (2.52) | 0.00 | 207 (1.47) | 216 (1.54) | 0.01 | 639 (2.23) | 630 (2.19) |
| Cerebrovascular procedure*,* n (%) | 5 (0.09) | 3 (0.05) | 0.01 | 17 (0.19) | 25 (0.28) | 0.02 | 0 (0.00) | 0 (0.00) | 0.00 | 22 (0.08) | 28 (0.10) |
| Congestive heart failure*,* n (%) | 1520 (27.18) | 1562 (27.93) | 0.02 | 707 (7.79) | 704 (7.76) | 0.00 | 863 (6.14) | 895 (6.37) | 0.01 | 3090 (10.76) | 3161 (11.01) |
| Peripheral vascular disease or surgery*,* n (%) | 221 (3.95) | 220 (3.93) | 0.00 | 1899 (20.93) | 1879 (20.71) | 0.01 | 245 (1.74) | 244 (1.74) | 0.00 | 2365 (8.24) | 2343 (8.16) |
| Atrial fibrillation*,* n (%) | 530 (9.48) | 584 (10.44) | 0.03 | 271 (2.99) | 292 (3.22) | 0.01 | 310 (2.21) | 341 (2.43) | 0.01 | 1111 (3.87) | 1217 (4.24) |
| Other cardiac dysrhythmia*,* n (%) | 242 (4.33) | 241 (4.31) | 0.00 | 377 (4.16) | 369 (4.07) | 0.00 | 561 (3.99) | 546 (3.89) | 0.01 | 1180 (4.11) | 1156 (4.03) |
| Cardiac conduction disorders*,* n (%) | 91 (1.63) | 97 (1.73) | 0.01 | 52 (0.57) | 49 (0.54) | 0.00 | 54 (0.38) | 50 (0.36) | 0.00 | 197 (0.69) | 196 (0.68) |
| Other CVD*,* n (%) | 647 (11.57) | 624 (11.16) | 0.01 | n/a | n/a | n/a | 521 (3.71) | 518 (3.69) | 0.00 | n/a | n/a |
| Oedema*,* n (%) | 144 (2.58) | 138 (2.47) | 0.01 | n/a | n/a | n/a | 52 (0.37) | 70 (0.50) | 0.02 | n/a | n/a |
| COPD*,* n (%) | 67 (1.20) | 65 (1.16) | 0.00 | 1219 (13.44) | 1241 (13.68) | 0.01 | 438 (3.12) | 459 (3.27) | 0.01 | 1724 (6.00) | 1765 (6.15) |
| Asthma*,* n (%) | 309 (5.53) | 266 (4.76) | 0.03 | 1989 (21.92) | 1964 (21.65) | 0.01 | 695 (4.95) | 699 (4.98) | 0.00 | 2993 (10.42) | 2929 (10.20) |
| Obstructive sleep apnoea*,* n (%) | 256 (4.58) | 279 (4.99) | 0.02 | 48 (0.53) | 50 (0.55) | 0.00 | 60 (0.43) | 70 (0.50) | 0.01 | 364 (1.27) | 399 (1.39) |
| Pneumonia*,* n (%) | 179 (3.20) | 187 (3.34) | 0.01 | 761 (8.39) | 761 (8.39) | 0.00 | 734 (5.22) | 794 (5.65) | 0.02 | 1674 (5.83) | 1742 (6.07) |
| Renal dysfunction (non-diabetic)*,* n (%) | 531 (9.50) | 510 (9.12) | 0.01 | n/a | n/a | n/a | 1333 (9.49) | 1316 (9.37) | 0.00 | n/a | n/a |
| Acute renal disease n (%) | 41 (0.73) | 40 (0.72) | 0.00 | 84 (0.93) | 83 (0.91) | 0.00 | 104 (0.74) | 100 (0.71) | 0.00 | 229 (0.80) | 223 (0.78) |
| Chronic renal insufficiency*,* n (%) | 323 (5.78) | 333 (5.95) | 0.01 | n/a | n/a | n/a | 238 (1.69) | 241 (1.72) | 0.00 | n/a | n/a |
| Chronic kidney disease*,* n (%) | 248 (4.43) | 274 (4.90) | 0.02 | 144 (1.59) | 150 (1.65) | 0.01 | 790 (5.62) | 767 (5.46) | 0.01 | 1182 (4.12) | 1191 (4.15) |
| Chronic kidney disease stage 3-4*,* n (%) | 22 (0.39) | 30 (0.54) | 0.02 | 27 (0.30) | 27 (0.30) | 0.00 | 322 (2.29) | 322 (2.29) | 0.00 | 371 (1.29) | 379 (1.32) |
| Hypertensive Nephropathy*,* n (%) | 3 (0.05) | 4 (0.07) | 0.01 | 63 (0.69) | 71 (0.78) | 0.01 | 231 (1.64) | 237 (1.69) | 0.00 | 297 (1.03) | 312 (1.09) |
| Miscellaneous renal insufficiency*,* n (%) | 218 (3.90) | 209 (3.74) | 0.01 | n/a | n/a | n/a | 147 (1.05) | 156 (1.11) | 0.01 | n/a | n/a |
| Liver disease*,* n (%) | 1072 (19.17) | 1038 (18.56) | 0.02 | 5512 (60.76) | 5520 (60.85) | 0.00 | 1739 (12.38) | 1768 (12.59) | 0.01 | 8323 (28.99) | 8326 (29.00) |
| Osteoarthritis*,* n (%) | 298 (5.33) | 270 (4.83) | 0.02 | 3380 (37.26) | 3391 (37.38) | 0.00 | 1949 (13.87) | 1918 (13.65) | 0.01 | 5627 (19.60) | 5579 (19.43) |
| Other arthritis, arthropathies and musculoskeletal pain*,* n (%) | 728 (13.02) | 674 (12.05) | 0.03 | 4082 (45.00) | 4012 (44.22) | 0.02 | 4266 (30.37) | 4234 (30.14) | 0.01 | 9076 (31.61) | 8920 (31.07) |
| Dorsopathies*,* n (%) | 1089 (19.47) | 1040 (18.60) | 0.02 | 5128 (56.53) | 5130 (56.55) | 0.00 | 3268 (23.26) | 3234 (23.02) | 0.01 | 9485 (33.03) | 9404 (32.75) |
| Fractures*,* n (%) | 193 (3.45) | 154 (2.75) | 0.04 | 791 (8.72) | 813 (8.96) | 0.01 | 573 (4.08) | 591 (4.21) | 0.01 | 1557 (5.42) | 1558 (5.43) |
| Falls*,* n (%) | n/a | n/a | n/a | n/a | n/a | n/a | 127 (0.90) | 131 (0.93) | 0.00 | n/a | n/a |
| Osteoporosis*,* n (%) | 233 (4.17) | 201 (3.59) | 0.03 | 1099 (12.11) | 1088 (11.99) | 0.00 | 230 (1.64) | 239 (1.70) | 0.00 | 1562 (5.44) | 1528 (5.32) |
| Hyperthyroidism*,* n (%) | 89 (1.59) | 94 (1.68) | 0.01 | 363 (4.00) | 385 (4.24) | 0.01 | 132 (0.94) | 120 (0.85) | 0.01 | 584 (2.03) | 599 (2.09) |
| Hypothyroidism*,* n (%) | 136 (2.43) | 123 (2.20) | 0.02 | 929 (10.24) | 943 (10.39) | 0.01 | 160 (1.14) | 171 (1.22) | 0.01 | 1225 (4.27) | 1237 (4.31) |
| Other disorders of thyroid gland*,* n (%) | 344 (6.15) | 341 (6.10) | 0.00 | 2262 (24.93) | 2327 (25.65) | 0.02 | 649 (4.62) | 661 (4.71) | 0.00 | 3255 (11.34) | 3329 (11.59) |
| Depression*,* n (%) | 190 (3.40) | 171 (3.06) | 0.02 | 1031 (11.36) | 1038 (11.44) | 0.00 | 540 (3.84) | 524 (3.73) | 0.01 | 1761 (6.13) | 1733 (6.04) |
| Anxiety*,* n (%) | 149 (2.66) | 114 (2.04) | 0.04 | 1588 (17.50) | 1619 (17.85) | 0.01 | 1022 (7.28) | 1039 (7.40) | 0.00 | 2759 (9.61) | 2772 (9.65) |
| Sleep disorder*,* n (%) | 953 (17.04) | 923 (16.51) | 0.01 | 1384 (15.26) | 1416 (15.61) | 0.01 | 1513 (10.77) | 1545 (11.00) | 0.01 | 3850 (13.41) | 3884 (13.53) |
| Dementia*,* n (%) | 62 (1.11) | 53 (0.95) | 0.02 | 197 (2.17) | 190 (2.09) | 0.01 | 202 (1.44) | 196 (1.40) | 0.00 | 461 (1.61) | 439 (1.53) |
| Delirium*,* n (%) | 75 (1.34) | 56 (1.00) | 0.03 | 15 (0.17) | 16 (0.18) | 0.00 | 8 (0.06) | 6 (0.04) | 0.01 | 98 (0.34) | 78 (0.27) |
| Psychosis*,* n (%) | 81 (1.45) | 71 (1.27) | 0.02 | 125 (1.38) | 118 (1.30) | 0.01 | 269 (1.91) | 265 (1.89) | 0.00 | 475 (1.65) | 454 (1.58) |
| Frailty*,* n (%) | 3345 (59.82) | 3322 (59.41) | 0.01 | n/a | n/a | n/a | n/a | n/a | n/a | n/a | n/a |
| Non-frailty*,* n (%) | 1889 (33.78) | 1745 (31.21) | 0.06 | n/a | n/a | n/a | n/a | n/a | n/a | n/a | n/a |
| **Laboratory values, mean (SD)** | | | | | | | | | | | |
| HbA_1c_ (%) | 8.08 (1.84) | 7.97 (1.81) | 0.06 | n/a | n/a | n/a | n/a | n/a | n/a | n/a | n/a |
| LDL level (mg/dl) | 114.57 (33.34) | 113.96 (37.30) | 0.02 | n/a | n/a | n/a | n/a | n/a | n/a | n/a | n/a |
| HDL level (mg/dl) | 48.39 (13.77) | 49.93 (12.89) | 0.12 | n/a | n/a | n/a | n/a | n/a | n/a | n/a | n/a |
| Total cholesterol (mg/dl) | 190.14 (41.55) | 192.53 (46.04) | 0.06 | n/a | n/a | n/a | n/a | n/a | n/a | n/a | n/a |
| Triglyceride level (mg/dl) | 191.53 (149.39) | 181.33 (130.70) | 0.07 | n/a | n/a | n/a | n/a | n/a | n/a | n/a | n/a |
| Creatinine (mg/dl) | 0.87 (0.45) | 0.88 (0.70) | 0.02 | n/a | n/a | n/a | n/a | n/a | n/a | n/a | n/a |
| BUN (mg/dl) | 15.76 (5.97) | 16.22 (7.91) | 0.07 | n/a | n/a | n/a | n/a | n/a | n/a | n/a | n/a |
| BNP (pg/mL) | 182.69 (288.07) | 188.87 (439.67) | 0.02 | n/a | n/a | n/a | n/a | n/a | n/a | n/a | n/a |
|  |  |  |  |  |  |  |  |  |  |  |  |
| **Prior concomitant use of other antidiabetic drugs** | | | | | | | | | | | |
| Number of antidiabetic substances at index date, mean (SD) | 2.05 (1.14) | 1.97 (1.10) | 0.07 | 2.10 (0.70) | 2.10 (0.68) | 0.00 | 2.62 (1.10) | 2.61 (1.03) | 0.01 | 2.34 | 2.32 |
| Naïve new use of antidiabetic drugs, ^§^ n (%) | 2223 (39.75) | 2417 (43.22) | 0.07 | 2306 (25.42) | 2311 (25.47) | 0.00 | 0 (0.00) | 0 (0.00) | 0.00 | 4529 (15.77) | 4728 (16.47) |
| Initiation of the study drug (empagliflozin/any SGLT-2 inhibitor/any DPP-4 inhibitor) as monotherapy*,* n (%) | 1707 (30.53) | 1868 (33.40) | 0.06 | 760 (8.38) | 737 (8.12) | 0.01 | 1623 (11.55) | 1679 (11.95) | 0.01 | 4090 (14.24) | 4284 (14.92) |
| Prior or current use of metformin (regardless of use of other antidiabetic agents)*,* n (%) | 1864 (33.33) | 1762 (31.51) | 0.04 | 7548 (83.20) | 7561 (83.34) | 0.00 | 10 408 (74.09) | 10 329 (73.53) | 0.01 | 19 820 (69.03) | 19 652 (68.45) |
| Current dual therapy with metformin (without use of other antidiabetic drugs)*,* n (%) | 508 (9.08) | 516 (9.23) | 0.00 | 899 (9.91) | 921 (10.15) | 0.01 | 4788 (34.08) | 4698 (33.44) | 0.01 | 6195 (21.58) | 6135 (21.37) |
| Prior or current use of exclusively metformin and no other antidiabetic drugs*,* n (%) | 744 (13.30) | 805 (14.40) | 0.03 | 3720 (41.01) | 3736 (41.18) | 0.00 | 2482 (17.67) | 2428 (17.28) | 0.01 | 6946 (24.19) | 6969 (24.27) |
| Concomitant initiation or current use of other antidiabetic drugs*,* n (%) | 1233 (22.05) | 1160 (20.74) | 0.03 | n/a | n/a | n/a | 7632 (54.33) | 7668 (54.58) | 0.01 | n/a | n/a |
| Past use of other antidiabetic drugs*,* n (%) | 1775 (31.74) | 1615 (28.88) | 0.06 | 7318 (80.67) | 7363 (81.16) | 0.01 | 9751 (69.41) | 9733 (69.28) | 0.00 | 18 844 (65.63) | 18 711 (65.17) |
| Sulphonylureas 2nd generation*,* n (%) | 567 (10.14) | 386 (6.90) | 0.12 | 3365 (37.1) | 3326 (36.66) | 0.01 | 5013 (35.68) | 5022 (35.75) | 0.00 | 8945 (31.15) | 8734 (30.42) |
| GLP-1 receptor agonists*,* n (%) | 673 (12.04) | 501 (8.96) | 0.10 | 39 (0.43) | 31 (0.34) | 0.01 | 272 (1.94) | 221 (1.57) | 0.03 | 984 (3.43) | 753 (2.62) |
| Thiazolidinediones*,* n (%) | 345 (6.17) | 268 (4.79) | 0.06 | 915 (10.09) | 914 (10.07) | 0.00 | 2340 (16.66) | 2379 (16.93) | 0.01 | 3600 (12.54) | 3561 (12.40) |
| Meglitinides*,* n (%) | 154 (2.75) | 146 (2.61) | 0.01 | 74 (0.82) | 74 (0.82) | 0.00 | 549 (3.91) | 573 (4.08) | 0.01 | 777 (2.71) | 793 (2.76) |
| Insulin*,* n (%) | 1844 (32.98) | 1768 (31.62) | 0.03 | 1269 (13.99) | 1272 (14.02) | 0.00 | 3854 (27.43) | 3828 (27.25) | 0.00 | 6967 (24.27) | 6868 (23.92) |
| Alpha-glucosidase inhibitors*,* n (%) | 436 (7.80) | 360 (6.44) | 0.05 | 347 (3.82) | 346 (3.81) | 0.00 | 1889 (13.45) | 1931 (13.75) | 0.01 | 2672 (9.31) | 2637 (9.18) |
| **Prior use of other drugs** | | | | | | | | | | | |
| ACE inhibitor*,* n (%) | 620 (11.09) | 623 (11.14) | 0.00 | 266 (2.93) | 283 (3.12) | 0.01 | 1,287 (9.16) | 1297 (9.23) | 0.00 | 2173 (7.57) | 2203 (7.67) |
| ARB*,* n (%) | 1788 (31.97) | 1718 (30.72) | 0.03 | 3958 (43.63) | 3958 (43.63) | 0.00 | 7336 (52.22) | 7286 (51.87) | 0.01 | 13 082 (45.56) | 12 962 (45.14) |
| Beta blocker*,* n (%) | 1309 (23.41) | 1353 (24.20) | 0.02 | 1406 (15.50) | 1400 (15.43) | 0.00 | 4754 (33.84) | 4762 (33.90) | 0.00 | 7469 (26.01) | 7515 (26.17) |
| Calcium channel blocker*,* n (%) | 1592 (28.47) | 1514 (27.07) | 0.03 | 1522 (16.78) | 1493 (16.46) | 0.01 | 6171 (43.93) | 6112 (43.51) | 0.01 | 9285 (32.34) | 9119 (31.76) |
| Thiazides*,* n (%) | 214 (3.83) | 199 (3.56) | 0.01 | 743 (8.19) | 733 (8.08) | 0.00 | 407 (2.90) | 411 (2.93) | 0.00 | 1364 (4.75) | 1343 (4.68) |
| Loop diuretics*,* n (%) | 874 (15.63) | 860 (15.38) | 0.01 | 539 (5.94) | 508 (5.60) | 0.01 | 1258 (8.96) | 1314 (9.35) | 0.01 | 2671 (9.30) | 2682 (9.34) |
| Other diuretics*,* n (%) | 588 (10.52) | 606 (10.84) | 0.01 | 300 (3.31) | 281 (3.10) | 0.01 | 613 (4.36) | 643 (4.58) | 0.01 | 1501 (5.23) | 1530 (5.33) |
| Nitrates*,* n (%) | 960 (17.17) | 1026 (18.35) | 0.03 | 645 (7.11) | 667 (7.35) | 0.01 | 1736 (12.36) | 1680 (11.96) | 0.01 | 3341 (11.64) | 3373 (11.75) |
| Other hypertension drugs*,* n (%) | 216 (3.86) | 214 (3.83) | 0.00 | 166 (1.83) | 158 (1.74) | 0.01 | 826 (5.88) | 843 (6.00) | 0.01 | 1208 (4.21) | 1215 (4.23) |
| Digoxin*,* n (%) | 69 (1.23) | 70 (1.25) | 0.00 | 109 (1.20) | 125 (1.38) | 0.02 | 224 (1.59) | 269 (1.91) | 0.02 | 402 (1.40) | 464 (1.62) |
| Antiarrhythmic drugs*,* n (%) | 246 (4.40) | 247 (4.42) | 0.00 | 75 (0.83) | 94 (1.04) | 0.02 | 459 (3.27) | 463 (3.30) | 0.00 | 780 (2.72) | 804 (2.80) |
| COPD or asthma medications*,* n (%) | 421 (7.53) | 372 (6.65) | 0.03 | 2203 (24.28) | 2200 (24.25) | 0.00 | 1936 (13.78) | 2028 (14.44) | 0.02 | 4560 (15.88) | 4600 (16.02) |
| Statin*,* n (%) | 2154 (38.52) | 2139 (38.25) | 0.01 | 6176 (68.08) | 6140 (67.68) | 0.01 | 9174 (65.30) | 9197 (65.47) | 0.00 | 17 504 (60.96) | 17 476 (60.87) |
| Other lipid-lowering drugs, excluding statins*,* n (%) | 733 (13.11) | 715 (12.79) | 0.01 | 1358 (14.97) | 1356 (14.95) | 0.00 | 10 000 (71.18) | 10 024 (71.36) | 0.00 | 12 091 (42.11) | 12 095 (42.13) |
| Antiplatelet*,* n (%) | 1496 (26.75) | 1504 (26.90) | 0.00 | 3319 (36.59) | 3282 (36.18) | 0.01 | 4643 (33.05) | 4599 (32.74) | 0.01 | 9458 (32.94) | 9385 (32.69) |
| Anticoagulants*,* n (%) | 407 (7.28) | 411 (7.35) | 0.00 | 198 (2.18) | 214 (2.36) | 0.01 | 315 (2.24) | 336 (2.39) | 0.01 | 920 (3.20) | 961 (3.35) |
| Heparin and other low-molecular weight heparins*,* n (%) | 1308 (23.39) | 1346 (24.07) | 0.02 | 345 (3.80) | 351 (3.87) | 0.00 | 531 (3.78) | 540 (3.84) | 0.00 | 2184 (7.61) | 2237 (7.79) |
| NSAIDs*,* n (%) | 1393 (24.91) | 1343 (24.02) | 0.02 | 6982 (76.96) | 7077 (78.01) | 0.03 | 9897 (70.45) | 9854 (70.15) | 0.01 | 18 272 (63.64) | 18 274 (63.65) |
| Bisphosphonates*,* n (%) | 94 (1.68) | 91 (1.63) | 0.00 | 289 (3.19) | 261 (2.88) | 0.02 | 49 (0.35) | 45 (0.32) | 0.01 | 432 (1.50) | 397 (1.38) |
| Opioids*,* n (%) | 106 (1.90) | 110 (1.97) | 0.01 | 4432 (48.85) | 4489 (49.48) | 0.01 | 1700 (12.10) | 1749 (12.45) | 0.01 | 6238 (21.73) | 6348 (22.11) |
| Antidepressants*,* n (%) | 184 (3.29) | 185 (3.31) | 0.00 | 1040 (11.46) | 1007 (11.10) | 0.01 | 1258 (8.96) | 1280 (9.11) | 0.01 | 2482 (8.64) | 2472 (8.61) |
| Antipsychotics*,* n (%) | 164 (2.93) | 149 (2.66) | 0.02 | 224 (2.47) | 234 (2.58) | 0.01 | 1222 (8.70) | 1267 (9.02) | 0.01 | 1610 (5.61) | 1650 (5.75) |
| Anticonvulsants*,* n (%) | 277 (4.95) | 284 (5.08) | 0.01 | 966 (10.65) | 979 (10.79) | 0.00 | 1134 (8.07) | 1180 (8.40) | 0.01 | 2377 (8.28) | 2443 (8.51) |
| Lithium*,* n (%) | 10 (0.18) | 15 (0.27) | 0.02 | 28 (0.31) | 22 (0.24) | 0.01 | 20 (0.14) | 23 (0.16) | 0.01 | 58 (0.20) | 60 (0.21) |
| Benzodiazepines*,* n (%) | 413 (7.39) | 402 (7.19) | 0.01 | 2433 (26.82) | 2482 (27.36) | 0.01 | 3772 (26.85) | 3739 (26.62) | 0.01 | 6618 (23.05) | 6623 (23.07) |
| Other anxiolytics/hypnotics*,* n (%) | 672 (12.02) | 659 (11.78) | 0.01 | 1225 (13.50) | 1262 (13.91) | 0.01 | 1860 (13.24) | 1870 (13.31) | 0.00 | 3757 (13.09) | 3791 (13.20) |
| Agents for dementia*,* n (%) | 1 (0.02) | 3 (0.05) | 0.02 | 544 (6.00) | 533 (5.88) | 0.01 | 296 (2.11) | 322 (2.29) | 0.01 | 841 (2.93) | 858 (2.99) |
| Antiparkinson agents*,* n (%) | 39 (0.70) | 39 (0.70) | 0.00 | 143 (1.58) | 134 (1.48) | 0.01 | 292 (2.08) | 305 (2.17) | 0.01 | 474 (1.65) | 478 (1.66) |
| **Health care resource use covariates** | | | | | | | | | | | |
| Combined comorbidity score, mean (SD) | 1.19 (1.83) | 1.15 (1.73) | 0.02 | 0.35 (1.03) | 0.32 (1.02) | 0.03 | 0.69 (1.36) | 0.69 (1.40) | 0.00 | 0.68 | 0.66 |
| Total number of distinct diagnosis codes, mean (SD) | 9.51 (6.84) | 9.10 (6.45) | 0.06 | 20.36 (12.05) | 20.57 (12.34) | 0.02 | 2.23 (1.46) | 2.24 (1.45) | 0.01 | 9.38 | 9.37 |
| Number of different medications, mean (SD) | 10.98 (9.86) | 10.67 (9.55) | 0.03 | n/a | n/a | n/a | 22.45 (14.57) | 22.64 (15.12) | 0.01 | n/a | n/a |
| Any hospitalization*,* n (%) | 2575 (46.05) | 2648 (47.35) | 0.03 | 1888 (20.81) | 2002 (22.07) | 0.03 | 1998 (14.22) | 1981 (14.10) | 0.00 | 6461 (22.50) | 6631 (23.09) |
| Any hospitalization within prior 30 days*,* n (%) | 1919 (34.32) | 1984 (35.48) | 0.02 | 390 (4.30) | 464 (5.11) | 0.04 | 74 (0.53) | 74 (0.53) | 0.00 | 2383 (8.30) | 2522 (8.78) |
| Any hospitalization during prior 31–365 days*,* n (%) | 927 (16.58) | 901 (16.11) | 0.01 | 1634 (18.01) | 1712 (18.87) | 0.02 | 1951 (13.89) | 1933 (13.76) | 0.00 | 4512 (15.71) | 4546 (15.83) |
| Number of hospitalizations, mean (SD) | 1.22 (0.62) | 1.19 (0.59) | 0.05 | 0.35 (1.00) | 0.40 (1.15) | 0.04 | 0.21 (0.70) | 0.20 (0.66) | 0.01 | 0.45 | 0.46 |
| Number of hospital days, mean (SD) | 10.91 (14.97) | 11.22 (14.13) | 0.02 | 2.71 (14.53) | 3.21 (17.12) | 0.03 | 1.40 (8.04) | 1.41 (6.98) | 0.00 | 3.67 | 3.89 |
| Number of emergency department visits, mean (SD) | 2.13 (3.53) | 2.22 (3.37) | 0.03 | 0.18 (0.65) | 0.19 (0.76) | 0.01 | 0.42 (1.07) | 0.42 (0.97) | 0.00 | 0.68 | 0.70 |
| Endocrinologist visit*,* n (%) | 492 (8.80) | 497 (8.89) | 0.00 | 1865 (20.56) | 1648 (18.17) | 0.06 | 6334 (45.09) | 6246 (44.46) | 0.01 | 8691 (30.27) | 8391 (29.22) |
| Number of endocrinologist visits, mean (SD) | 7.15 (6.77) | 6.80 (8.22) | 0.05 | 0.82 (1.96) | 0.71 (1.92) | 0.05 | 4.27 (6.01) | 4.24 (6.08) | 0.01 | 3.74 | 3.62 |
| Internal medicine/family medicine visits*,* n (%) | 2636 (47.14) | 2486 (44.46) | 0.05 | 8872 (97.80) | 8812 (97.13) | 0.04 | 10 635 (75.70) | 10 589 (75.38) | 0.01 | 22 143 (77.12) | 21 887 (76.23) |
| Number of internal medicine/family medicine visits, mean (SD) | 8.61 (11.00) | 8.07 (11.21) | 0.05 | 10.96 (10.31) | 10.80 (9.51) | 0.02 | 7.38 (10.09) | 7.45 (9.76) | 0.01 | 8.75 | 8.63 |
| Cardiologist visit*,* n (%) | 1623 (29.02) | 1764 (31.55) | 0.05 | 1566 (17.26) | 1318 (14.53) | 0.07 | 4345 (30.93) | 4331 (30.83) | 0.00 | 7534 (26.24) | 7413 (25.82) |
| Number of cardiologist visits, mean (SD) | 8.26 (12.21) | 7.18 (8.55) | 0.10 | 0.69 (1.83) | 0.58 (1.70) | 0.06 | 2.36 (4.57) | 2.35 (4.53) | 0.00 | 2.98 | 2.73 |
| Electrocardiogram*,* n (%) | 3211 (57.42) | 3378 (60.41) | 0.06 | 3687 (40.64) | 3572 (39.37) | 0.03 | 5335 (37.98) | 5388 (38.35) | 0.01 | 12 233 (42.61) | 12 338 (42.97) |
| Number electrocardiograms received, mean (SD) | 2.01 (2.18) | 1.85 (1.75) | 0.08 | 0.68 (1.19) | 0.67 (1.20) | 0.01 | 0.65 (1.18) | 0.65 (1.13) | 0.01 | 0.92 | 0.89 |
| Number of HbA_1c_ tests ordered | 6.48 (6.23) | 4.96 (4.50) | 0.28 | n/a | n/a | n/a | 3.69 (1.84) | 3.69 (1.91) | 0.00 | n/a | n/a |
| Number of glucose tests ordered, mean (SD) | n/a | n/a | n/a | n/a | n/a | n/a | 5.20 (3.31) | 5.22 (3.19) | 0.01 | n/a | n/a |
| Number of lipid tests ordered, mean (SD) | n/a | n/a | n/a | n/a | n/a | n/a | 6.11 (3.54) | 6.08 (3.59) | 0.01 | n/a | n/a |
| Number of creatinine tests ordered, mean (SD) | 5.00 (4.24) | 4.83 (4.67) | 0.04 | n/a | n/a | n/a | 3.12 (2.28) | 3.09 (2.31) | 0.01 | n/a | n/a |
| Number of BUN tests ordered, mean (SD) | 4.69 (4.30) | 4.63 (4.25) | 0.01 | n/a | n/a | n/a | 0.81 (1.56) | 0.82 (1.46) | 0.00 | n/a | n/a |
| Number of tests for microalbuminuria, mean (SD) | 4.31 (4.02) | 4.08 (4.66) | 0.05 | n/a | n/a | n/a | 1.15 (1.20) | 1.14 (1.24) | 0.01 | n/a | n/a |
| Total cost of care, USD, mean (SD) **^¶^** | 5633.63  (8 885.32) | 5502.99  (8 811.72) | 0.01 | 1203.48 (2438.41) | 1454.98 (4121.75) | 0.07 | 1778.99  (2679.96) | 1702.15  (3745.62) | 0.02 | 8616.10 (n/a) | 8660.12 (n/a) |
| Total cost of care, local currency, mean (SD) | 612 038.03 (965 30 0.85) | 597 844.83 (957 305.49) | 0.01 | 1444176  (2926092) | 174 5976  (4946100) | 0.07 | 55 789.07 (84 043.49) | 53 379.47 (117 462.61) | 0.02 | n/a | n/a |
| Inpatient cost, USD, mean (SD) | 8040.85  (10 384.09) | 8 077.36  (9 674.22) | 0.00 | 558.59 (2106.84) | 769.37 (3727.26) | 0.07 | 408.83  (1810.56) | 431.52  1784.49 | 0.01 | 9008.27 (n/a) | 9278.25 (n/a) |
| Inpatient cost, local currency, mean (SD) | 873 557.42 (1 128 127.16) | 877 523.97 (1 051 007.38) | 0.00 | 67 0308  (2528208) | 92 3244  (4472712) | 0.07 | 12 821.03 (56 779.01) | 13 532.55 (55 961.62) | 0.01 | n/a | n/a |
| Total non-CV-related inpatient cost, USD, mean (SD) | 4563.51  (5 062.04) | 5004.36  (6 618.74) | 0.07 | 202.56 (982.18) | 293.87 (2001.92) | 0.06 | 262.25  1390.74 | 281.02  1437.26 | 0.01 | 5028.32 (n/a) | 5579.25 (n/a) |
| Total non-CV-related inpatient cost, local currency, mean (SD) | 495 779.25 (549 939.96) | 543 673.19 (719 060.26) | 0.07 | 243072  (1178616) | 352644  (2402304) | 0.06 | 8224.17 (43 613.56) | 8812.69 (45 072.38) | 0.01 | n/a | n/a |
| Total outpatient cost, USD, mean (SD) | 2250.54  (3 514.39) | 1990.64  (4245.22) | 0.07 | 630.98 (824.26) | 662.24 (1123.93) | 0.03 | 1126.66  1612.84 | 1086.98  3053.79 | 0.02 | 4008.18 (n/a) | 3739.86 (n/a) |
| Total outpatient cost, local currency, mean (SD) | 244 499.03 (381 803.51) | 216 263.22 (461 201.00) | 0.07 | 757176  (989112) | 794688  (1348716) | 0.03 | 35 331.91 (50 578.71) | 34 087.61 (95 766.81) | 0.02 | n/a | n/a |
| Total CV-related outpatient cost, USD, mean (SD) | 1905.91  (2090.67) | 1606.92  (2724.87) | 0.12 | 179.48 (318.21) | 187.83 (583.22) | 0.02 | 165.14  404.35 | 162.29  392.68 | 0.01 | 2250.53 (n/a) | 1957.04 (n/a) |
| Total CV-related outpatient cost, local currency, mean (SD) | 207 057.94 (227 130.75) | 174 575.39 (296 029.49) | 0.12 | 215376  (381852) | 225396  (699864) | 0.02 | 5178.65 (12 680.47) | 5089.51 (12 314.54) | 0.01 | n/a | n/a |
| Total non-CV-related outpatient cost, USD, mean (SD) | 1 243.94  (2 925.50) | 1 224.28  (3 443.93) | 0.01 | 451.49 (720.68) | 474.41 (876.11) | 0.03 | 961.52  1573.43 | 924.68  3028.74 | 0.01 | 2656.95 (n/a) | 2623.37 (n/a) |
| Total non-CV-related outpatient cost, local currency, mean (SD) | 135 141.25 (317 826.25) | 133 005.90 (374 148.47) | 0.01 | 541788  (864816) | 569292  (1051332) | 0.03 | 30 153.26 (49342.66) | 28 998.10 (94 981.27) | 0.01 | n/a | n/a |
| Total pharmacy cost, USD, mean (SD) | 1 346.74  (3 057.06) | 1 232.07  (3 851.77) | 0.03 | n/a | n/a | n/a | 243.50  465.34 | 183.65  296.26 | 0.15 | n/a | n/a |
| Total pharmacy cost, local currency, mean (SD) | 146 309.66 (332 119.47) | 133 852.37 (418 456.39) | 0.03 | n/a | n/a | n/a | 7636.13 (14 592.97) | 5759.31 (9290.62) | 0.15 | n/a | n/a |
| Total pharmacy cost for antidiabetic medications, USD, mean (SD) | 509.32  (1 086.58) | 347.78  (1 726.51) | 0.11 | n/a | n/a | n/a | 96.90  363.16 | 56.36  143.87 | 0.15 | n/a | n/a |
| Total pharmacy cost for antidiabetic medications, local currency, mean (SD) | 55332.88 (118 046.19) | 37 783.25 (187 568.48) | 0.11 | n/a | n/a | n/a | 3038.89 (11 388.72) | 1767.60 (4511.88) | 0.15 | n/a | n/a |
| Total pharmacy cost for non-antidiabetic medications, USD, mean (SD) | 1 072.23  (2 921.44) | 1 072.06  (3 663.71) | 0.00 | n/a | n/a | n/a | 128.38  205.79 | 106.74  203.53 | 0.11 | n/a | n/a |
| Total pharmacy cost for non-antidiabetic medications, local currency, mean (SD) | 116487.49 (317 385.78) | 116 469.10 (398 025.00) | 0.00 | n/a | n/a | n/a | 4026.13 (6453.60) | 3347.45 (6382.81) | 0.11 | n/a | n/a |

ACE, angiotensin converting enzyme; ARB, angiotensin receptor blocker; ASD, absolute standardized difference; BUN, blood urea nitrogen; CABG, coronary artery bypass grafting; COPD, chronic obstructive pulmonary disease; CVD, cardiovascular disease; DPP-4, dipeptidyl peptidase-4; GLP, glucagon-like peptide; HbA1c, glycated haemoglobin; HDL, high-density lipoprotein; LDL, low-density lipoprotein; NT-proBNP, N-terminal B-type natriuretic peptide; PTCA, Percutaneous transluminal coronary angioplasty; TIA, transient ischaemic attack

^†^Household income was divided into three groups (lower 30%, middle 40%, and upper 30%) based on health insurance premium

^‡^Based on ICD-10 codes

^§^Defined as patients without any use of glucose-lowering medications during the 12 months prior to cohort entry

**^¶^**Exchange rates calculated as of 31^st^ July 2019

**Supplementary Table 3. Definitions of outcomes**

|  | **Japan** | | **South Korea** |  | **Taiwan** | | |
| --- | --- | --- | --- | --- | --- | --- | --- |
| **Outcome** | **Definition** | **ICD 10 code** | **Definition** | **ICD 10 code** | **Definition** | **ICD 9 code** | **ICD 10 code** |
| Hospitalization for heart failure^a^ | HHF-broad: any inpatient visit with a HF diagnosis code associated  HHF-specific: inpatient HF diagnosis that either required the most health care resources, triggered the hospitalization, or was coded as main disease on the DPC hospital claim. | I11.0, I13.0, I13.2, I50 | HHF-specific and HHF-broad both included primary diagnosis and any diagnosis of hospitalization | I50 | HHF-broad: hospitalization with any diagnosis of HF  HHF-specific: hospitalization when HF was the primary diagnosis at hospitalization | 428.x, 398.91, 402.01,  402.11, 402.91, 404.01,  404.11, 404.91, 404.03,  404.13, 404.93 | I50, I09.81, I11.0,  I13.0, I13.2 |
| All-cause mortality | Recorded from DPC records |  | Linked to national death registry |  | Linked to national death registry |  |  |
| ESRD | Any diagnosis or procedure associated with  Health care encounters, including  hospitalizations and specialist outpatient  and primary care encounters | eGFR<15, at least 2  measurements separated by ≥30  days (≤12 months)  OR  ≥2 of the following diagnosis or  procedure codes (either in/ outpatient),  separated by ≥30 days  Diagnoses: disease code  8842116, 8847583, 8848103Procedures: procedure code  140057810, 140057910, 140058010, 140059310, 140059410, 140059510, 140058110, 140058210, 140058310, 140058410, 140058510, 140058610OR  Kidney transplant, defined as ≥1  of the following diagnosis or  procedure codes (in/out-patient):  Diagnoses: disease code  8847618, 9968003, 8847643, 8847671, 8846302, 8835575, 8835577,  8846303  Procedures: procedure code  150338610 | Any diagnosis associated with health care  encounters, including hospitalizations and  specialist outpatient and primary care  encounters | Diagnoses: N18  Procedures: O702x, O707x  OR  Diagnoses: Z94.0, Z99.2  Procedures: R3280 | Any diagnosis associated with health care encounters, including hospitalizations and specialist outpatient and primary care  encounters | eGFR < 15, at least 2  measurements separated  by at least 30 days (but  no more than 12  months)  OR  ≥2 of the following  diagnosis or procedure  codes (either inpatient  or outpatient), separated  by at least 30 days  Diagnoses: V56.0, V56.8, V45.1  Procedures: 39.95,  54.98  OR  Kidney transplant,  defined as ≥1 of the  following diagnosis or  procedure codes  (inpatient or outpatient):  Diagnoses: V42.0,  996.81  Procedures: 55.6x | eGFR < 15, at least 2  measurements  separated by at least  30 days (but no more  than 12 months)  OR  ≥2 of the following  diagnosis or  procedure codes  (either inpatient or  outpatient), separated  by at least 30 days  Diagnoses: N18.5, N18.6, Z49.31,  Z49.32, Z99.2  Procedures:  OR  Kidney transplant,  defined as ≥1 of the  following diagnosis or  procedure codes  (inpatient or  outpatient)  Diagnoses: Z48.22,  Z94.0, T86.1 |

^a^includes fatal and non-fatal cases

**Supplementary Table 4. Reasons for censoring**

|  | **Empagliflozin** | | | | | | **DPP4-inhibitor** | | | | | |
| --- | --- | --- | --- | --- | --- | --- | --- | --- | --- | --- | --- | --- |
|  | **Number of patients** | **Patients with event** | **Patients censored due to death** | **Patients censored due to index drug*** | **Patients censored due to end of data availability due to end of study period** | **Patients censored due to end of data availability due to other reasons** | **Number of patients** | **Patients with event** | **Patients censored due to death** | **Patients censored due to index drug*** | **Patients censored due to end of data availability due to end of study period** | **Patients censored due to end of data availability due to other reasons** |
| **As treated analysis** | | | | | | | | | | | | |
| **HHF broad** | | | | | | | | | | | | |
| Japan | 5592 | 181 | 12 | 3084 | 2315 | 0 | 5592 | 216 | 23 | 3336 | 2017 | 0 |
| South Korea | 9072 | 77 | 11 | 1959 | 7025 | 0 | 9072 | 100 | 25 | 2300 | 6647 | 0 |
| Taiwan | 14048 | 113 | 64 | 6902 | 6969 | 0 | 14048 | 126 | 98 | 7691 | 6133 | 0 |
| **All-cause mortality** | | | | | | | | | | | | |
| Japan | 5592 | 20 | 0 | 3192 | 2380 | 0 | 5592 | 30 | 0 | 3472 | 2090 | 0 |
| South Korea | 9072 | 18 | 0 | 1980 | 7074 | 0 | 9072 | 33 | 0 | 2342 | 6697 | 0 |
| Taiwan | 14048 | 76 | 0 | 6961 | 7011 | 0 | 14048 | 110 | 0 | 7761 | 6177 | 0 |
| **End-stage renal disease** | | | | | | | | | | | | |
| Japan | 5592 | 6 | 19 | 3191 | 2376 | 0 | 5592 | 17 | 30 | 3460 | 2085 | 0 |
| South Korea | 9072 | 0 | 18 | 1980 | 7074 | 0 | 9072 | 9 | 33 | 2339 | 6691 | 0 |
| Taiwan | 14048 | 21 | 75 | 6954 | 6998 | 0 | 14048 | 52 | 110 | 7739 | 6147 | 0 |
| **Intent to treat analysis** | | | | | | | | | | | | |
| **HHF broad** | | | | | | | | | | | | |
| Japan | 5592 | 317 | 43 | 0 | 4478 | 754 | 5592 | 356 | 45 | 0 | 4344 | 847 |
| South Korea | 9072 | 100 | 23 | 0 | 8949 | 0 | 9072 | 126 | 50 | 0 | 8896 | 0 |
| Taiwan | 14048 | 183 | 125 | 0 | 13740 | 0 | 14048 | 242 | 214 | 0 | 13592 | 0 |
| **All-cause mortality** | | | | | | | | | | | | |
| Japan | 5592 | 63 | 0 | 0 | 4741 | 788 | 5592 | 61 | 0 | 0 | 4644 | 887 |
| South Korea | 9072 | 37 | 0 | 0 | 9035 | 0 | 9072 | 65 | 0 | 0 | 9007 | 0 |
| Taiwan | 14048 | 153 | 0 | 0 | 13895 | 0 | 14048 | 260 | 0 | 0 | 13788 | 0 |
| **End-stage renal disease** | | | | | | | | | | | | |
| Japan | 5592 | 11 | 62 | 0 | 4731 | 788 | 5592 | 37 | 60 | 0 | 4609 | 886 |
| South Korea | 9072 | 0 | 37 | 0 | 9035 | 0 | 9072 | 13 | 64 | 0 | 8995 | 0 |
| Taiwan | 14048 | 37 | 152 | 0 | 13859 | 0 | 14048 | 86 | 255 | 0 | 13707 | 0 |

**The follow-up time will be censored in occurrence of discontinuation of the initial drug use, switch to any other study drug (empagliflozin, any SGLT-2 inhibitor, any DPP-4 inhibitor (including drugs that are marketed locally and are not study drugs in this multi-country studies)), initiation of concomitant use of empagliflozin/SGLT-2 inhibitor and a DPP-4 inhibitor use (including drugs that are marketed locally and are not study drugs in this multi-country study) or drugs within the same class (i.e. two SGLT-2 inhibitors or two DPP-4 inhibitors) either as free or fixed dose combination. So if we know that the index drug use has come to an end or concomitant use has been started, this is the reason for censoring in the AT analysis. This cause of censoring will not be used in ITT analysis.

**Supplementary Table 5. Risk of outcomes in 1:1 propensity score-matched patients (empagliflozin 10 mg subgroup)^†^**

|  | **Empagliflozin 10 mg** | **DPP-4 inhibitor** | **HR (95% CI)** |
| --- | --- | --- | --- |
| **Risk of event in 1:1 propensity score-matched patients** | **N events (rate per 1000 patient-years)** | **N events (rate per 1000 patient-years)** |  |
| Patients, n | 21 542 | 21 542 |  |
| HHF specific (sensitivity analysis)^‡^ | 137 (12.52) | 159 (14.94) | 0.85 (0.67,1.07) |
| HHF-specific (strict definition)^§^ | 75 (6.84) | 77 (7.22) | 0.99 (0.72, 1.36) |
| HHF-broad | 309 (28.37) | 373 (35.29) | 0.83 (0.71, 0.96) |
| All-cause mortality | 92 (8.37) | 111 (10.37) | 0.85 (0.64, 1.12) |
| End stage renal disease | 23 (1.10) | 41 (3.84) | 0.63 (0.37, 1.06) |

HHF, hospitalization for Heart Failure; HR, hazard ratio

^†^In Japan and South Korea, patients were censored if titrated to a different empagliflozin dose; in Taiwan patients initiating on to empagliflozin 10 mg/day were considered to be part of that subgroup regardless of future dose adjustment.

^‡^HF as primary diagnosis code or main reason of admission for Japan and Taiwan, and hospitalization with any diagnosis code of HF in South Korea.

^§^HF as primary diagnosis code or main reason of admission in all countries.
